# Optical metalenses: fundamentals, dispersion manipulation, and applications

**DOI:** 10.1007/s12200-022-00017-4

**Published:** 2022-05-18

**Authors:** Yongli He, Boxiang Song, Jiang Tang

**Affiliations:** 1 Wuhan National Laboratory for Optoelectronics, Huazhong University of Science and Technology, Wuhan, 430074 China; 2 School of Optical and Electronic Information, Huazhong University of Science and Technology, Wuhan, 430074 China

**Keywords:** Metasurfaces, Metalenses, Flat optics, Nanophotonics, Chromatic and monochromatic aberrations

## Abstract

Metasurfaces, also known as 2D artificial metamaterials, are attracting great attention due to their unprecedented performances and functionalities that are hard to achieve by conventional diffractive or refractive elements. With their sub-wavelength optical scatterers, metasurfaces have been utilized to freely modify different characteristics of incident light such as amplitude, polarization, phase, and frequency. Compared to traditional bulky lenses, metasurface lenses possess the advantages of flatness, light weight, and compatibility with semiconductor manufacture technology. They have been widely applied to a range of scenarios including imaging, solar energy harvesting, optoelectronic detection, etc. In this review, we will first introduce the fundamental design principles for metalens, and then report recent theoretical and experimental progress with emphasis on methods to correct chromatic and monochromatic aberrations. Finally, typical applications of metalenses and corresponding design rules will be presented, followed by a brief outlook on the prospects and challenges of this field.

**Graphical abstract:**

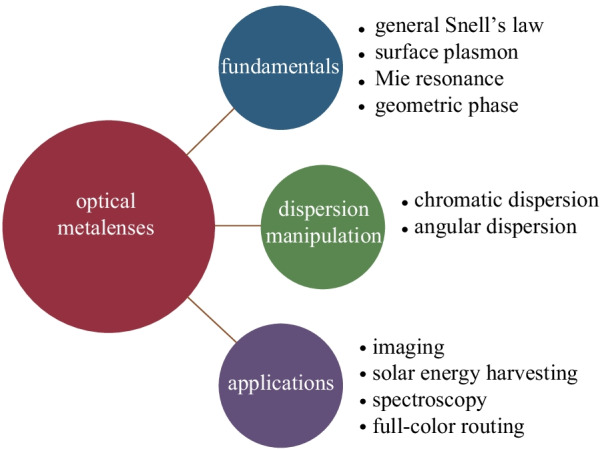

## Introduction

Recently, optical metalenses have received increasing attention for their various applications in solar energy harvesting [1, 2], imaging, optoelectronic devices, etc. Conventional optical devices, such as convex lenses and concave mirrors, focus light based on geometric optics. Such lenses and mirrors have been used in various applications in our daily lives for hundreds of years. However, these conventional optical devices are usually bulky, heavy, and costly due to the diffraction limits (D. L.) requirement and long optical path for phase manipulation, which has made them unsuitable in modern electronic and photonic systems with demands for miniaturization and integration. In addition, the fabrication of these refractive lenses and reflective mirrors is based on traditional technology and molding, which further limits their development.

Metasurfaces are composed of 2D periodic arrays of subwavelength-spaced optical scatterers (metallic or dielectric) that can modify different characteristics of incident light and exhibit new optical properties [3–6]. Based on the design of optical scatters, the metasurfaces can couple and reradiate incident light, in different ways. For example, the scattered light may have locally shifted phase profile and therefore reshaped wavefront, different polarization direction and angular distribution or rearranged spectral contents. By proper adjustment on the location, spacing, and geometric parameters of the unit-cell, the wavefront and other characteristics of the reflected or transmitted light can be engineered for various applications. As a result, different conventional optical device functionalities including light focusing have already been realized using metasurfaces. The arrangement of unit-cells across metasurfaces at the subwavelength scale enables the control of light properties with high spatial resolution. Typically, the height of unit-cells is also at subwavelength scale with planar form factor. The subwavelength nanostructures of metasurfaces can be manufactured on a large scale at a low cost using standard nanofabrication and microfabrication techniques. Furthermore, the subwavelength spacing in metasurfaces avoids the spurious diffraction orders, which exist commonly in general diffractive optical elements (DOEs). These spurious diffraction orders reduce the efficiency of light focusing and lead to various unexpected effects such as halos, virtual focal spots, and ghost images when applied to imaging-related applications. It should be noted that subwavelength DOEs such as blazed gratings can circumvent spurious diffraction orders. However, their performances and functionalities degrade significantly especially when the numerical apertures (NAs) increase [7, 8]. The high versatility and flexibility of metasurface design provide opportunities for various proper designs to overcome these problems and outperform conventional DOEs. In that sense, the combination of low cost, high-efficiency, compact structure and planar platform, large numerical aperture, wide bandwidth, and reduced aberrations can be realized simultaneously for light focusing application by using metasurfaces. This has attracted many researchers from diverse disciplines and backgrounds. With their contributions, various development and breakthrough in this field have been reported. In this review, we focus on recent developments of metasurface lenses.

In the past few years, the developments and advances in microfabrication and nanofabrication have paved the road for novel optical manipulation of optical wavefronts in metasurfaces and led to blossoming of research of metalenses [8–10]. First, we discuss the fundamental design rule and theoretical analysis of metalenses. Metasurfaces focus light by introducing a novel method of wavefront shaping within the compact and planar platform. The flat or planar optics forms the basis of metalenses and further improvements in various applications. Plasmonic optical metasurfaces are the early demonstration of generalized flat optics including metalenses [11, 12]. The unit-cells of plasmonic metasurfaces are deep-subwavelength-spaced metallic diffractive elements, which show severe dissipative loss in the visible and near-infrared (NIR) range and therefore low efficiency of focusing. By switching from plasmonic metasurfaces to high index all-dielectric metasurfaces, the efficiency of focusing has improved significantly. Next, we discuss recent progress in the development of all-dielectric metalenses. Reflective and transmissive metalenses are compared in their applications in devices and miniaturized systems. High efficiency, large NAs, correction of optical aberrations, control on chromatic dispersion and angular response can be realized by advances in the design and fabrication of metasurfaces. Metalenses, along with their superior advantages, can be used in diverse applications such as imaging, optoelectronic devices, and solar energy collection. We conclude by summarizing the significance of metalenses and by outlooking the potentials and challenges in this field of science and technology.

## Fundamental design principles and experimental demonstrations

The variation in effective permittivity, permeability and refractive index of unit-cells in metasurfaces introduces minimal propagation phase shift due to the subwavelength thickness. Meanwhile, the tailored surface impedance including phase, amplitude, phase, and polarization incurred during reflection and transmission upon light incidence into metasurfaces, is critical for the design of metalenses. Abrupt changes in optical properties are introduced in metasurfaces and these changes are widely applied instead of propagation effects to focus light [13–15]. These abrupt and controllable changes of optical properties can be engineered by accurately adjusting the materials and parameters of each unit-cell, which have a variety of forms, ranging from metallic (plasmonic) or dielectric nanostructures [16–19], apertures forms inside continuous films [20, 21] and corresponding multi-layer structures [22]. The arrays of subwavelength-spaced unit-cells can have spatially varying structural features or material compositions, and they therefore modify a spatially distributed optical response (e.g. amplitude, phase, and polarization) and form a reshaped wavefront on demand.

**Fig. 1:**
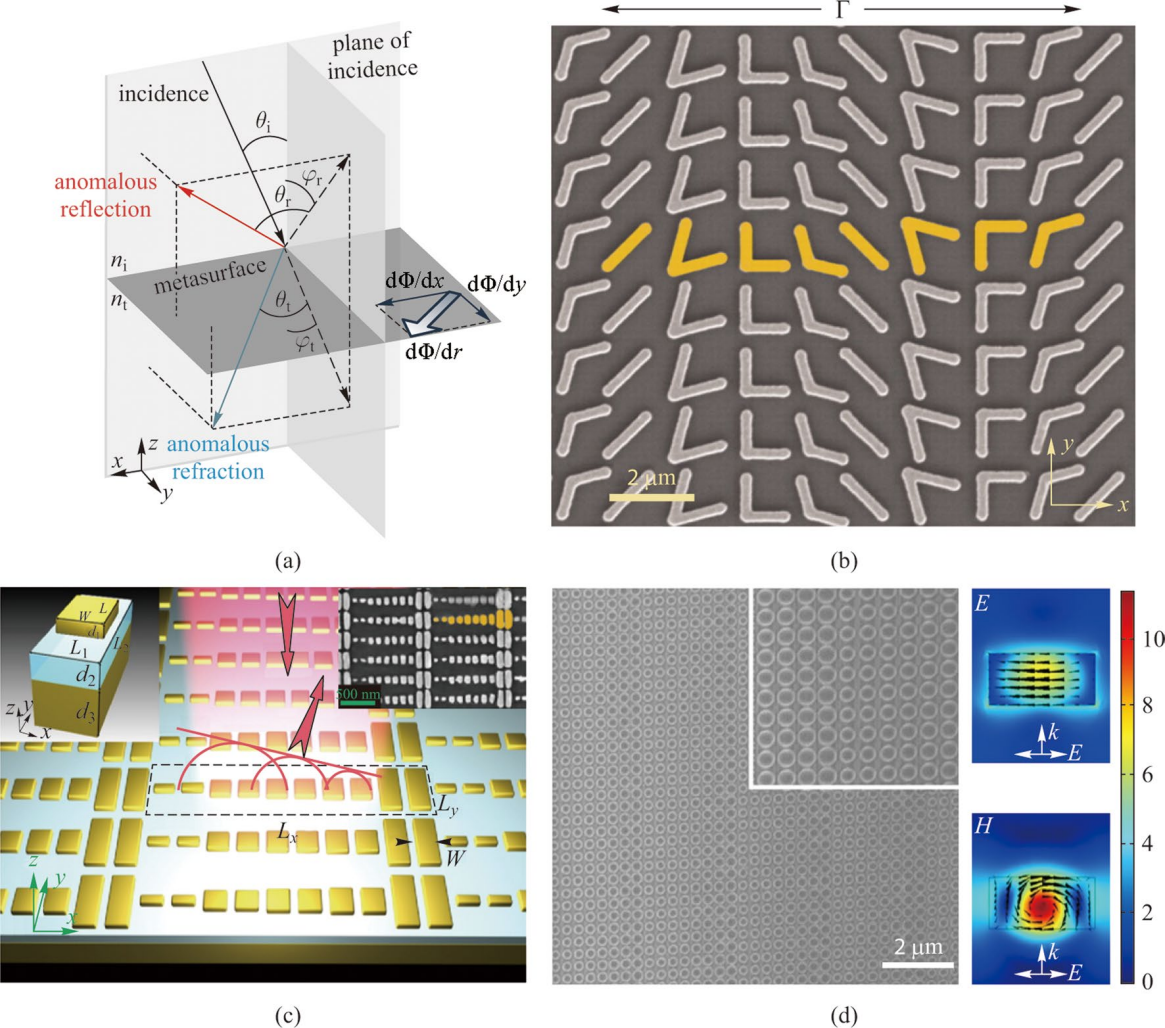
Pioneer works demonstrating the fundamental design rules. **a** Schematics of the generalized Snell’s law of reflection and refraction. The gradient of phase shift d*Φ/*d*r* at the interface offers an effective wavevector that can bend reflected and transmitted light in designed directions. **b** Scanning electron microscope (SEM) image of the plasmonic metasurface with V-shaped optical antennas. **c** Schematic of the reflect-array metasurface with gold patch antennas separated from a gold substrate by a dielectric spacer with subwavelength thickness. The left inset shows a schematic of an individual unit-cell, and the right inset is the corresponding SEM image of the metasurface. **d** SEM image of a dielectric metasurface Huygens’ beam deflector and the corresponding simulated field distributions. **a** Reprinted with permission of IOP Publishing, from Ref. [23]; permission conveyed through Copyright Clearance Center, Inc. **b** Reprinted from Ref. [13]. Copyright 2011, The American Association for the Advancement of Science. **c** Reprinted with permission from Ref. [24]. Copyright 2012, American Chemical Society. **d** Reprinted with permission from Ref. [25]. Copyright 2015, WILEY‐VCH Verlag GmbH & Co. KGaA, Weinheim

Upon incidence onto an interface between two homogeneous media with distinct refractive indices, the electromagnetic wave is split into reflected beam and transmitted beam. Conventionally, the reflection and transmission directions and coefficients are given by the Fresnel equations and Snell’s law with respect to the continuity of the field components at the interface. The resonant excitation of each unit-cell across the metasurfaces modify the boundary conditions dramatically. The abrupt phase shift introduced by the resonant unit-cells ranges from –π to π, depending on the difference between the frequency of incident wave and the unit-cells’ resonant frequency. The polarization state can be also altered by anisotropic unit-cells. The reflection and refraction remain in conventional direction if the phase shift is uniform across the metasurface. Metasurfaces offer freedom to engineer each unit-cell and can further exhibit spatial phase variation with subwavelength resolution. Therefore, the direction of reflected and transmitted wave propagation and the shape of wavefront can be tailored at will. A set of generalized laws of reflection and refraction are given to depict the effect from metasurfaces [23]:

1 $$\begin{array}{c}{n}_{t}\text{sin}{\theta }_{t}-{n}_{i}\text{sin}{\theta }_{i}=\frac{1}{{k}_{0}}\frac{\text{d}\Phi }{\text{d}x},\end{array}$$

2 $$\begin{array}{c}\text{cos}{\theta }_{t}\text{sin}{\varphi }_{t}=\frac{1}{{{n}_{t}k}_{0}}\frac{\text{d}\Phi }{\text{d}y},\end{array}$$

3 $$\begin{array}{c}\text{sin}{\theta }_{r}-\text{sin}{\theta }_{i}=\frac{1}{{{n}_{i}k}_{0}}\frac{\text{d}\Phi }{\text{d}x},\end{array}$$

4 $$\begin{array}{c}\text{cos}{\theta }_{r}\text{sin}{\varphi }_{r}=\frac{1}{{{n}_{r}k}_{0}}\frac{\text{d}\Phi }{\text{d}y},\end{array}$$

where the definition of the angles and phase gradients is shown in Fig. 1a. The generalized laws are the guide for design of metalenses, and indicate that the reflected and transmitted waves can propagate in required directions according to a tailored interfacial phase gradient across the metasurfaces. The superposition of spherical waves scattered from each unit-cell, with spacing much smaller than the wavelength, forms the reflected and refracted waves, following Huygens’ principle.

The experimental demonstration of optical manipulation by metasurfaces relies on the abrupt phase shift generated in the unit-cells. For metallic unit-cells, the incident light energy will be translated into internal charge oscillation known as surface plasmon. The mismatch between the incident frequency and the intrinsic resonant frequency of corresponding unit-cell will introduce a phase variation to the scattered light. On the other hand, the dielectric unit-cells introduce phase variation to the scattered light with Mie resonance, which involves the establishment of standing wave patterns in the dielectric unit-cells.

As shown in Fig. 1b, metasurfaces with V-shaped metallic unit-cells were first used to demonstrate the generalized optical laws [13]. These gradient phased metasurfaces can function in a similar way to blazed gratings [26–29] but with better broadband performances of flat optical components. Reflect-arrays were also used to demonstrate the generalized optical laws in metasurfaces. Given the example shown in Fig. 1c, the metallic patch antennas are separated from a metallic substrate by a thin dielectric layer [24]. The phase variation in reflect-array metasurfaces is created by the coupling between antennas and their image dipoles in the metallic substrates, which achieves a phase coverage of 2π. Despite the success in the lower frequency range, the metallic Huygens’ metasurfaces suffer from severe material losses in the optical frequency domain. In the past few years, low-loss high-index dielectric materials have been applied in metasurface research as unit-cells in order to address the efficiency issue posed in metallic metasurfaces. Dielectric Huygens’s metasurfaces have been proposed [25] where two longitudinal resonance modes with electric and magnetic dipole at the same frequency are supported (Fig. 1d).

In particular, metasurfaces focus light by converting the incident wavefronts into spherical ones, which converge at a focus *f* from the metasurfaces. To realize that, the metasurface phase profile $\varphi (R)$ should be accurately digitized and implemented as a hyperbolic, which ensures a diffraction-limited focus spot [12],

5 $$\varphi \left(R\right)=-\frac{2\uppi }{\lambda }\left(\sqrt{{R}^{2}{+f}^{2}}-f\right),$$

where *R* is the radial position on the metasurface, and *f* is the focal length. Equation (5) represents the requirement that normally incident light of wavelength $\lambda$ should converge at the focus in phase.

**Fig. 2:**
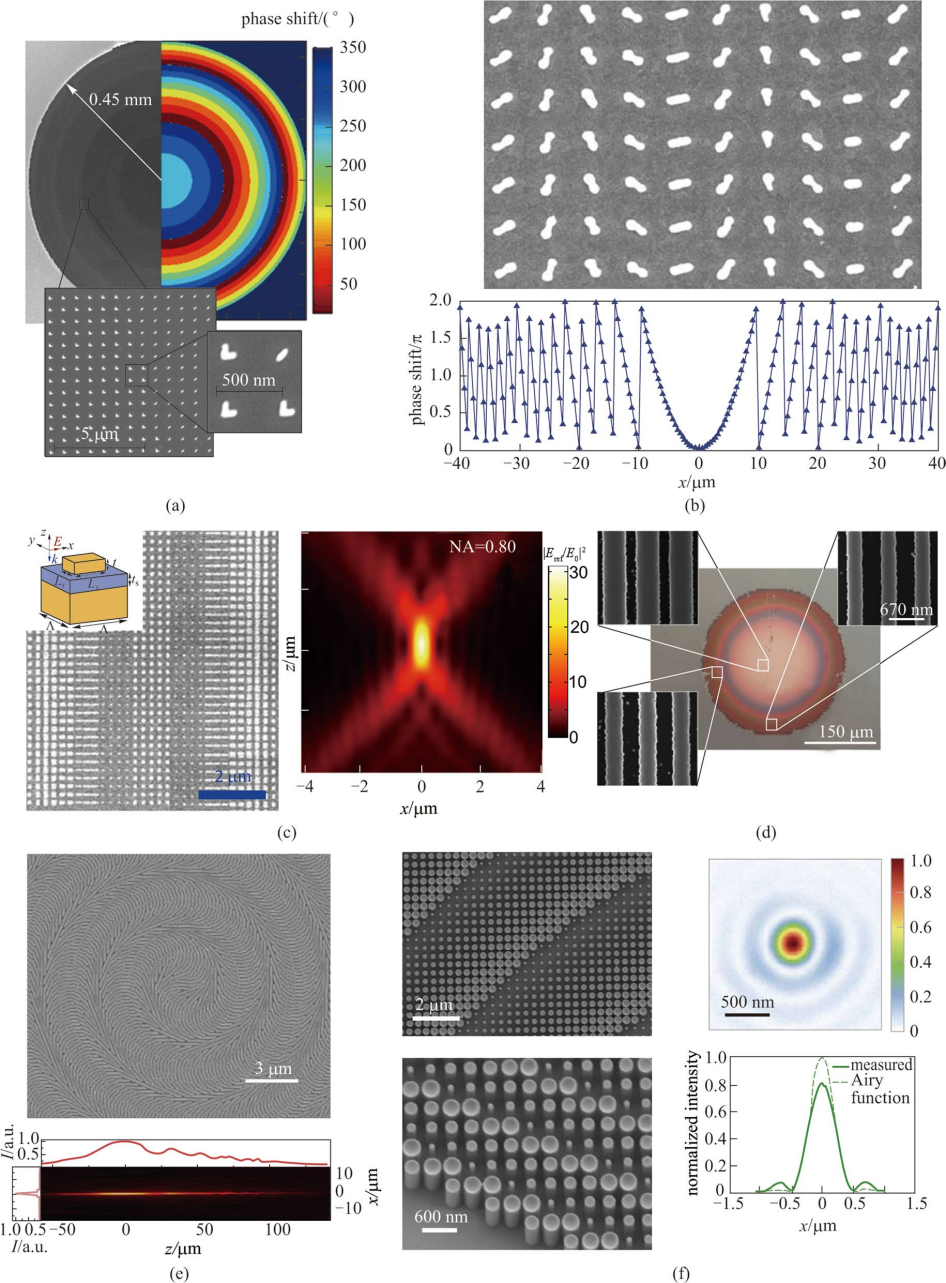
Typical examples of 2π phase coverage realization in light-concentrating metasurfaces. **a** SEM image of the metasurface with V-shaped antennas and the corresponding phase shift profile. **b** SEM image and expected phase discontinuity of a plasmonic metasurface on an ITO-coated glass substrate with positive polarity for incident lights with right circular polarization. **c** SEM image and measured intensity distribution near the focus of a cylindrical metalens with 0.8 NA. The inset shows the schematic of an individual unit cell. **d** Optical micrograph and SEM images of a high-contrast grating metalens. **e** SEM image of a geometric phase metasurface with dielectric microbars and corresponding measured intensity distribution along the propagation direction. **f** Top-view and side-view SEM images of the polarization insensitive metasurfaces, and the measured focal profile and corresponding horizontal cut of the focal spot at 532 nm. **a** Reprinted with permission from Ref. [12]. Copyright 2012, American Chemical Society. **b** Reprinted with permission from Ref. [34]. Copyright 2012, Chen et al. **c** Reprinted with permission from Ref. [35]. Copyright 2013, American Chemical Society. **d** Reprinted with permission from Ref. [36]. Copyright 2010, Springer Nature. **e** Reprinted with permission from Ref. [37]. Copyright 2014, The American Association for the Advancement of Science. **f** Reprinted with permission from Ref. [38]. Copyright 2016, American Chemical Society

In early works, light focusing achieved by plasmonic metasurfaces has been demonstrated via amplitude modulation and appropriate spatial design of plasmonic apertures [30–32]. To obtain a diffraction-limited focus with high focusing efficiency requires 2π phase coverage with identical scattering amplitude for all unit-cells across the metasurfaces. For the transmissive metasurface light concentrator, it requires that unit-cells should have negligible optical absorption loss and strongest forward scattering. Using V-antennas that have proved generalized laws of reflection and refraction [13, 33], Aieta and coworkers demonstrated flat metalenses that focused light in the NIR range without spherical aberration [12] (Fig. 2a). In the following works, focusing efficiency was improved by increasing the number of layers as the scattering efficiency of the individual antenna layer was low [39]. On the other hand, unit-cells with identical sizes can vary phases via their rotation and cover the 2π phase range, which is known as Pancharatnam-Berry (PB) phase or geometric phase (Fig. 2b) [34, 40, 41]. The PB phase based metasurface will switch the polarization of incident light to the orthogonal direction, which is their intrinsic property. In the NIR and mid-infrared range, reflective metasurfaces made of reflecting arrays of antennas separated from a metallic mirror by a dielectric spacer are demonstrated to focus light with incident polarization preserved (Fig. 2c) [35]. Despite the major development of the plasmonic metasurfaces, the intrinsic absorption loss of metal at visible and NIR range poses a fundamental limit on the efficiency that plasmonic metasurfaces can achieve. Researchers have proposed all-dielectric metasurfaces to solve this problem. Using dielectric phase shifters as unit-cells to focus light, people have developed various technological advances. These dielectric metasurfaces can manipulate the wavefronts with negligible absorption loss. By adjusting the geometrical parameters of unit-cells, the phase coverage can reach the required 2π range, mostly via propagation phase. The spatial discretization of the phase profile, which is defined as the center-to-center distance *U* between adjacent unit-cells, should satisfy the required sampling criterion

6 $$\begin{array}{c}U<\frac{\lambda }{2\text{NA}}.\end{array}$$

This is required to achieve diffraction-limited focusing. While the wavelength becomes shorter or the NA becomes higher, the smaller value of *U* poses higher requirements in the fabrication process. On the other hand, as the adjacent unit-cells get closer, reduction of optical confinement and greater near-field coupling become severe. For focusing at visible wavelength range and with large NA, the value of *U* should be accordingly small and therefore result in a quasi-adiabatic change in the size of unit-cells across the metasurface. High-index dielectrics can satisfy this criterion with enhanced light confinement and reduced near-field coupling. Silicon-based metasurfaces are widely used platforms to focus light particularly in the NIR range based on their large refractive index and mature fabrication technology [36, 37, 42–45]. Reflective dielectric metasurface with one-dimensional amorphous silicon ($\alpha$ Si) gratings in which the period and duty cycle were gradually altered from the center to the edge (Fig. 2d) [36] was demonstrated as a light concentrator. Since the unit-cells in these metasurfaces are asymmetric, the light concentrator is polarization sensitive. Instead of reflection, light focusing via a Si based transmissive metalens using PB phase was also achieved (Fig. 2e) [37]. In the visible range where the optical loss of silicon is significant, dielectric material that is transparent in this region is used, such as silicon nitride [46] and titanium dioxide [28]. The circular or fourfold symmetry in individual dielectric unit-cell is required to build polarization-independent metalenses. Polarization insensitive light focusing in the visible range was demonstrated by Khorasaninejad et al. (Fig. 2f) [38]. The atomic-layer-deposited (ALD) titanium dioxide circular unit-cells provided full 2π phase coverage with subwavelength spatial resolution. The metasurface exhibited diffraction-limited light focusing with high NA and efficiency at designated visible wavelengths.

## Chromatic dispersion manipulation

The bandwidth of operation is the wavelength range of the light that can be focused by the metasurface into a designated focal area. Chromatic dispersion is one of the intrinsic characteristics of metasurfaces, since the phase profile provided in Eq. (5) is dependent on the incident light wavelength. This chromatic dispersion strongly limits the further applications of metalenses, including colorful imaging and solar energy collection. In this section, we review the recent progress in solving the problem of chromatic dispersion in metasurfaces and manipulating the phase profile over a continuous wavelength range.

### Multiwavelength

**Fig. 3:**
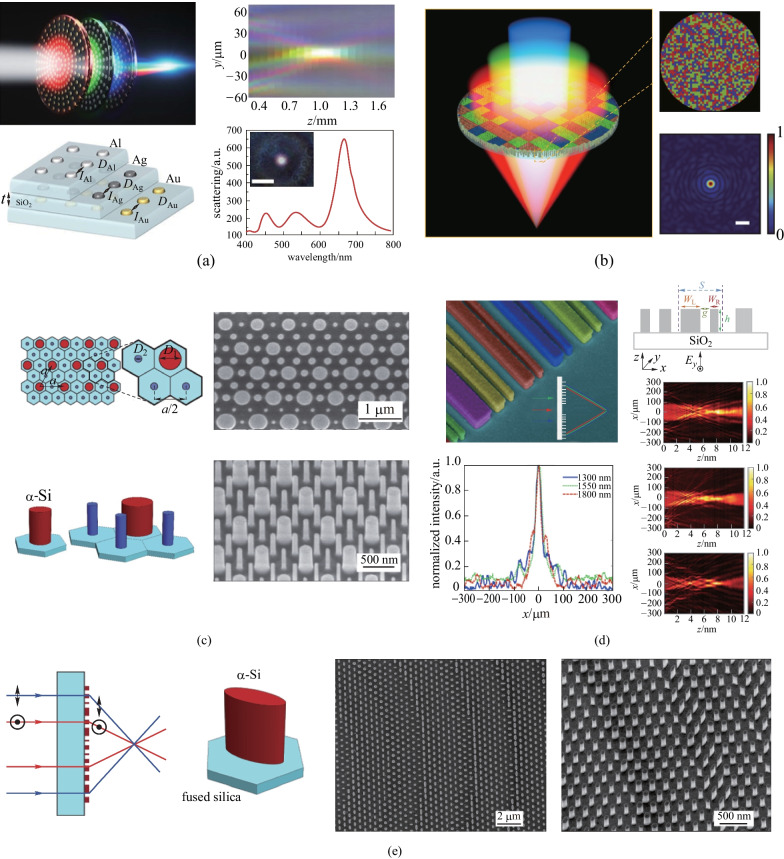
Multiwavelength achromatic metalens. **a** Tandem-stacked multilayered plasmonic multiwavelength metalens designed using frequency-dependent scatterers. **b** Multiwavelength metalens multiplexed by segmentation. **c** Multiwavelength polarization-insensitive metalenses with unit cells composed of meta-atoms. **d** Metasurfaces consisting of coupled rectangular dielectric resonators as unit-cells to introduce the desired phase profiles simultaneously at three wavelengths (1300, 1550, and 1800 nm) with dispersion compensation. **e** Birefringent metalenses with elliptical meta-atoms designed to focus light with two different wavelengths and orthogonal polarizations. **a** Reprinted from permission from Ref. [47]. Copyright 2017, Avayu et al. **b** Reprinted with permission from Ref. [48], IOP Publishing, permission conveyed through Copyright Clearance Center, Inc. **c** Reprinted with permission from Ref. [52]. Copyright 2016, The Optical Society. **d** Reprinted with permission from Ref. [49]. Copyright 2015, American Chemical Society. **e** Reprinted with permission from Ref. [54]. Copyright 2016, The Optical Society

In imaging applications, many have proposed multiwavelength achromatic metalenses in the visible spectrum range. They applied dispersion engineering of the nanostructures in metasurfaces in order to improve the performances of metalenses simultaneously at a set of discrete wavelengths. Avayu et al. applied tandem-stacked multilayered plasmonic metasurfaces to demonstrate triple wavelength multiplexing in the visible range (Fig. 3a) [47]. Three layers of metasurfaces made of metallic nano-discs with different materials were closely stacked together. Each layer had a different resonant wavelength which corresponded to red, green, and blue respectively. However, low efficiency and fabrication difficulty limit its further applications. The spatial multiplexing metasurfaces were designed based on dividing one metasurface into macroscopic segments with functionality corresponding to different wavelengths [48, 50, 51]. Yuan et al. demonstrated an example of this device, shown in Fig. 3b [48]. The phase profile was segmented to accommodate red, green, and blue wavelengths, respectively. To obtain optical focal performances at different wavelengths, randomized distribution of the segments is preferred. However, the multiwavelength operation in these devices sacrificed the focusing efficiency due to the parallel architecture. Also, the incorporation of phase profile designated to distinct wavelengths would incur imperfect phase and elevate the background. Another design for multiwavelength operation is based on the unit-cells (i.e., meta-molecule) composed of multiple nanoposts (meta-atoms) which behave as individual scatterers with weak coupling in between (Fig. 3c) [49, 52, 53]. Each individual meta-atom is designed to provide the required phase shift independently at corresponding wavelengths. In that sense, meta-molecules with more different nanoposts can provide extra parameters to tune the combination of two-phase changes at two different wavelengths with better accuracy [52].

Different from the methods mentioned above based on combining or interleaving different macroscopic sections or individual scatterers designed for corresponding wavelengths, the meta-atoms can be engineered at both structure and material level to accommodate for multiwavelength light focusing. With appropriate designs, the meta-atoms can demonstrate desired phase variation at different wavelengths. Aieta et al. used coupled rectangular dielectric resonators as unit-cells to introduce the desired phase profiles simultaneously at three wavelengths (1300, 1550, and 1800 nm) with dispersion compensation [53]. By using this approach, they achieved a cylindrical achromatic metalens with the focusing efficiency being 15%, 10%, and 21% at the three corresponding wavelengths. Additionally, the focal spot is close to the diffraction limit for a NA of 0.05 (Fig. 3d) [49]. Another design is based on the anisotropic property of elliptical silicon nanoposts. Since the elliptical geometry offers two independent parameters that can be utilized to demonstrate distinct phase control upon incident light, the birefringent metalenses were proposed to focus light with two different wavelength and orthogonal polarizations (Fig. 3e) [54–56]. This approach is efficient for double wavelength operation and can provide large effective NA. However, it has limited applications as the inherent achromatism principle is valid for only two different wavelengths and is sensitive to incident polarizations.

### Continuous wavelength range

The multiwavelength metalens is a significant step to realization of broadband operation with high focus efficiency. However, the ideal phase shift profile should satisfy a wavelength-dependent function that compensates for the dispersion in a continuous wavelength range. In that case, new approaches or design principles are demanded for the improvement of broadband operation in metasurfaces.

**Fig. 4:**
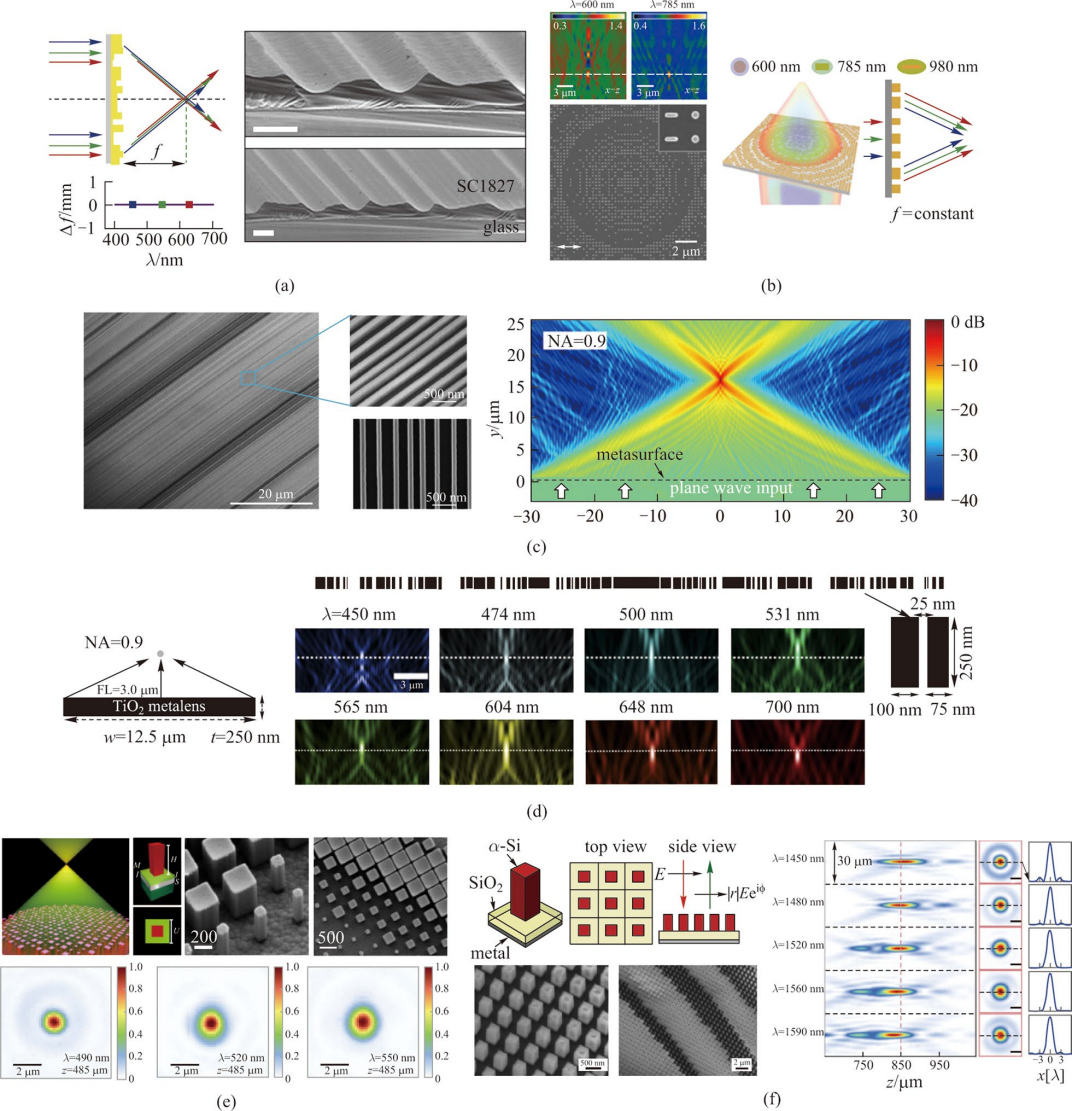
Broadband metalenses designed with different optimization algorithms. **a** Achromatic focusing at three discrete wavelengths (460, 540, and 620 nm) by chromatic-corrected diffractive metalenses optimized with direct-binary-search algorithm. **b** Multiwavelength achromatic lenses designed with lattice evolution algorithm. **c** Metalens with operation wavelengths from 580 to 700 nm range using topology optimization. **d** Achromatic metalenses with large NA via inverse design approach utilizing plane-wave mode decomposition. **e** An achromatic metalens over a continuous visible wavelength range made of TiO_2_ nanopillars, a dielectric spacer, and a metallic back reflector. **f** Dispersion-engineered metasurfaces over the wavelength range of 1450 to 1590 nm with minimized chromatic dispersion. **a** Reprinted with permission from Ref. [63]. Copyright 2016, Wang et al. **b** Reprinted with permission from Ref. [64]. Copyright 2016, American Chemical Society. **c** Reprinted with permission from Ref. [65]. Copyright 2019, Phan et al. **d** Reprinted with permission from Ref. [66]. Copyright 2020, The Optical Society. **e** Reprinted with permission from Ref. [67]. Copyright 2017, American Chemical Society. **f** Reprinted with permission from Ref. [68]. Copyright 2017, The Optical Society

Topology optimization is an effective method for metasurface design capable of multiwavelength or continuous wavelength operation [57, 58]. Conventionally, the study of light in those subwavelength photonic structures has relied on intuition-based rules and knowledge. However, computational inverse design is a powerful algorithmic technique that has developed in recent years for topology optimization [59–62]. It is used to discover optical structures based on desired functional characteristics, including broadband light focusing. Various inverse designed optical metasurfaces based on different optimization algorithms have been demonstrated to focus light at multiple wavelengths or in a continuous wavelength range with required focusing properties. Direct-binary-search algorithm was applied by Wang et al. to design chromatic-corrected diffractive metalenses composed of linear grooves [63]. While the widths are fixed according to the resolution of fabrication procedure, the heights of the grooves are optimized by this algorithm, which is basically a perturbation-based iterative process for optimal achromatic properties. The metasurfaces fabricated according to this design demonstrated achromatic focusing at three discrete wavelengths (460, 540, and 620 nm) with 20% to 25% focusing efficiency (Fig. 4a). Benefiting from the optimized design, the nanostructures of the metasurfaces are as simple and straightforward as can be fabricated with this one-step greyscale lithography. Hu et al. proposed a lattice evolution algorithm based on finite-difference time-domain (FDTD) simulations for the design of flat achromatic metalenses at 600, 785, and 980 nm (Fig. 4b) [64]. The scattering optical fields from each subwavelength plasmonic nanoparticle can be simulated by this method, and therefore optimization for multiwavelength performances can be realized by tuning the arrangement of the phase units on a discrete square lattice. This lattice evolution algorithm is essentially a multi-objective optimization algorithm capable of extending the working wavelengths to deep ultraviolet and near infrared by the replacement of unit-cell materials. Fan and his coworkers presented a computationally efficient approach for topology optimization [65]. They approximated the desired phase profile with a series of linear segments to solve the dilemma between high simulation load and broadband performances. The subwavelength scale 1D scattering gratings designed and fabricated according to this algorithm can strongly scatter light at the desired angle and phase. In that sense, they demonstrated a metalens operating from 580 to 700 nm range while 0.8 NA and 50% averaged efficiency were achieved within this range (Fig. 4c). By breaking the local-periodicity assumptions of the unit cells in metasurfaces, Chung and Miller demonstrated achromatic metalenses with large NA via an inverse design approach utilizing plane-wave mode decomposition [66]. In this work, they displayed broadband high-NA structures for the visible spectrum in 2D devices (Fig. 4d). Due to the incompleteness of the unit cell basis, they demonstrated the computational upper bounds on the maximal focusing efficiency. Actually, these optimization approaches merely provided the design approximation for multiwavelength operation or broadband achromatism as a compromise to the intrinsic chromatic dispersion in metasurfaces. Thus this problem is not solved by the optimization algorithm in principle, and the overall focusing efficiency of algorithm-optimized metasurfaces is not high over a large wavelength range.

Combining the conventional design strategy based on well-known physical properties of materials and structures with the inverse design optimization algorithm, researchers proposed a better solution to chromatic dispersion. Reflective metallic mirrors that can introduce large amplitude and wide phase coverage can be applied together with dielectric scatterers to improve achromatic performances. Capasso and coworkers sandwiched a thin silicon dioxide (SiO_2_) layer between square titanium dioxide (TiO_2_) nanopillar arrays and metallic mirrors to demonstrate an achromatic metalens over a continuous visible wavelength range (Fig. 4e) [67]. The square cross-section of the nanopillars ensures the maximal phase coverage as well as the polarization-insensitive performances. The optimization algorithm was then utilized to determine the geometric parameters and distributions of the dielectric nanopillars. In this way, they minimized the difference between the implemented and desired phase profile simultaneously within the demanded wavelength range from 490 to 550 nm. In the infrared range, Faraon and his collaborators showed an achromatic reflective metalens with a similar principle [68]. The square amorphous silicon nanoposts were fabricated on the SiO_2_ spacer layer on top of an aluminum reflector (Fig. 4f). The metasurface exhibits around 50% efficiency over the wavelength range of 1450 to 1590 nm with minimized chromatic dispersion. They pointed out that independent control over phase and dispersion of meta-atoms provides additional freedoms that can be applied in optimization algorithm to deal with chromatic dispersion in metasurfaces over continuous wavelength regimes.

Wang et al. proposed a novel design principle to eliminate chromatic dispersion in broadband operation [69]. They illustrated reflective broadband achromatic metalenses and gradient metasurfaces in the infrared range of 1200 to 1680 nm. However, it is only effective for circularly polarized incident light. According to the general metasurface phase profile shown in Eq. (5), it is a function of wavelength *λ* and can be expressed as $\varphi \left(R, \lambda \right)$, which can be divided into two components

7 $$\begin{array}{c}\varphi \left(R, \lambda \right)=\varphi \left(R, {\lambda }_{\text{max}}\right)+\Delta \varphi \left(R, \lambda \right).\end{array}$$

**Fig. 5:**
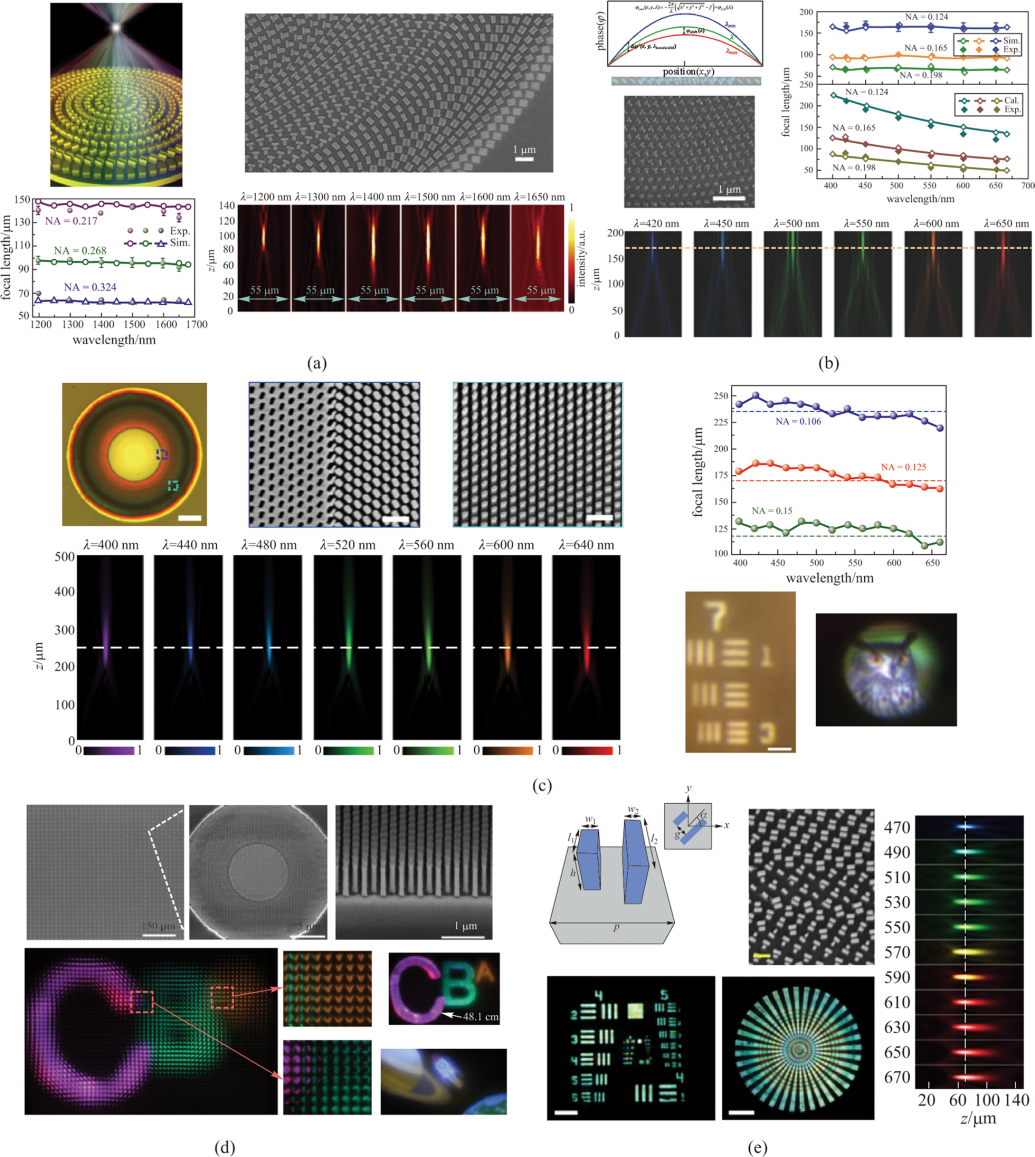
Dispersion manipulation based on compensation phase. **a** Reflective broadband achromatic metalenses in the infrared range of 1200 to 1680 nm realized by Au integrated-resonant unit elements and new design principles. **b** Visible range achromatic metalenses operating from 400 to 667 nm achieved by Al integrated-resonant unit elements. **c** Transmissive achromatic metalens operating from 400 to 600 nm made of GaN nanopillars and nanoholes. **d** A full-color light field camera composed of multiple achromatic GaN metalens arrays. **e** A transmissive broadband achromatic metalens operating in the visible from 470 to 670 nm made of coupled TiO_2_ nanofins for each unit cell. **a** Reproduced with permission from Ref. [69]. Copyright 2017, Wang et al. **b** Reproduced with permission from Ref. [73]. Copyright 2018, WILEY‐VCH Verlag GmbH & Co. KGaA, Weinheim. **c** Reprinted with permission from Ref. [70]. Copyright 2018, Springer Nature Customer Service Centre GmbH: Springer Nature, Nature Nanotechnology. **d** Reprinted with permission from Ref. [71]. Copyright 2019, Springer Nature Customer Service Centre GmbH: Springer Nature, Nature Nanotechnology. **e** Reprinted with permission from Ref. [72]. Copyright 2018, Springer Nature Customer Service Centre GmbH: Springer Nature, Nature Nanotechnology

$\varphi \left(R, {\lambda }_{\text{max}}\right)$ is the basic phase profile corresponding to the largest desired working wavelength, which is not related to the incident wavelength. $\Delta \varphi \left(R, \lambda \right)$ is the compensation term for the phase variance as the wavelength changes, which is considered to be the chromatic aberration phase. In their work, the basic phase profile was achieved by geometric phase modulation in each meta-atom which is guided by their orientation on the metasurfaces. The acquirement of geometric phase is the origin of the limitation on the circularly polarized incident light in this work. For the chromatic aberration phase, the basic unit cell consisting of Au nanorods, SiO_2_ spacer and Au back reflector was employed to obtain a compensation phase that had linear form versus 1/*λ* (Fig. 5a). While the geometric phase was achieved by the orientation of unit cells, the chromatic aberration phase was realized in the integrated resonance from the unit cell architecture. Therefore, two different phase components can directly add up together without interference to fulfill this wavelength-dependent total phase profile. A continuous examination of the constant focal length with different NAs was performed to verify the broadband light focusing in this metalens. With a similar design, aluminum was used to replace Au for visible range broadband light focusing, since aluminum has higher plasmon frequency and lower loss in the visible range. The achromatic metalenses design by this method is effective from 400 to 667 nm, as shown in Fig. 5b [73]. Other than the reflective broadband metalenses, the transmissive devices are highly desirable in many practical situations such as imaging applications. With the similar design of integrated resonant unit cells, gallium nitride (GaN) was applied to achieve the transmissive achromatic metalens from 400 to 600 nm [70]. In the visible range, GaN exhibits high transmission efficiency as well as high refractive index that makes it an ideal material to demonstrate the chromatic phase compensation. Increased thickness of the nanostructures will directly provide large phase compensation to cover the full 2π range owing to the weak optical coupling in the high index scatterers. As the key factor in controlling the phase shift, the effective index is tuned by the duty cycle. Full-color imaging, as well as video with extraordinary resolution down to 2.19 µm, was displayed with this transmissive achromatic metalens in the visible spectrum (Fig. 5c). Lin et al. demonstrated a full-color light field camera with a diffraction-limited 1.95 µm resolution by combining multiple achromatic GaN metalenses into a $60\times 60$ array (Fig. 5d) [71].

Another approach to tailor the chromatic dispersion of phase shift in metasurfaces was performed by Capasso and his collaborators to achieve a transmissive broadband achromatic metalens in the visible range from 470 to 670 nm [72]. In their method, the general phase profile in Eq. (5) can be expanded as a Taylor series near a design frequency ${\omega }_{\text{d}}$ as

8 $$\varphi \left(R,\omega \right)=\varphi \left(R,{\omega }_{\text{d}}\right)+{\left.\frac{\partial \varphi \left(R,\omega \right)}{\partial \omega }\right|}_{\omega ={\omega }_{\text{d}}}\left(\omega -{\omega }_{\text{d}}\right)+{\left.\frac{{\partial }^{2}\varphi \left(R,w\right)}{2\partial {\omega }^{2}}\right|}_{\omega ={\omega }_{\text{d}}}{\left(\omega -{\omega }_{\text{d}}\right)}^{2}+\dots .$$

$\varphi \left(R,{\omega }_{\text{d}}\right)$ is the required relative phase for the determined frequency ${\omega }_{\text{d}}$, while the higher-order derivatives $\frac{\partial \varphi \left(R,\omega \right)}{\partial \omega }$ and $\frac{{\partial }^{2}\varphi \left(R,w\right)}{\partial {\omega }^{2}}$ are the relative group delay and group delay dispersion that determine the chromatic dispersion. They utilized two TiO_2_ nanofins in close proximity as coupled waveguides for each unit cell. By accurate control of the geometry of the nanofin pair, the group delay and group delay dispersion could be engineered to minimize the chromatic dispersion. Similarly, the basic phase shift was achieved through a frequency-independent geometric phase that was determined by the rotation angle of coupled nanofins. Independent control of phase and dispersion was therefore achieved in this design and the broadband achromatic metalens was demonstrated from 470 to 670 nm (Fig. 5e). As the geometric phase was adopted in this design, the achromatic metalens could only work with circularly polarized incident light.

**Fig. 6:**
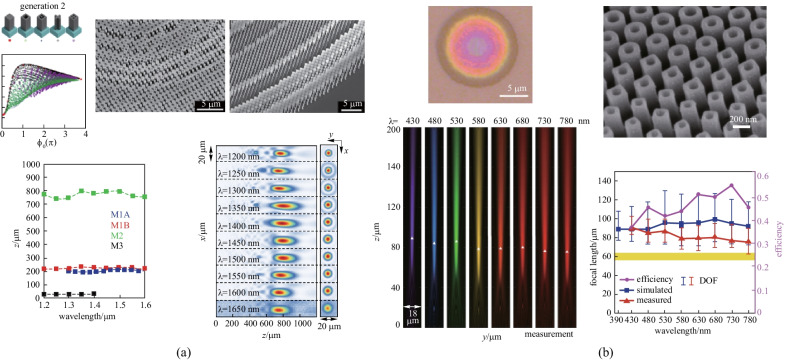
Polarization insensitive broadband metalens with isotropic symmetry. **a** Broadband achromatic metalenses made of libraries of meta-units with complex cross-sectional geometries to provide diverse phase dispersions for arbitrary polarization state from 1200 to 1650 nm. **b** A silicon nitride metalens in the visible region with zero effective material dispersion and an effective achromatic refractive index distribution from 430 to 780 nm. **a** Reprinted with permission from Ref. [74]. Copyright 2018, Shrestha et al. **b** Reprinted with permission from Ref. [75]. Copyright 2019, Fan et al.

In many practical applications, polarization-insensitive broadband achromatic metasurfaces are preferred, given that the sunlight is unpolarized. Shrestha et al. proved a polarization-independent continuous diffraction-limited achromatic light focusing across a broad near-infrared bandwidth at transmissive mode [74]. They developed a design methodology and created libraries of meta-units (unit-cells) with complex cross-sectional geometries to provide diverse phase dispersions for arbitrary incident illumination. The fundamental mathematical equations were derived, governing the tradeoffs between phase dispersion and achievable lens parameters, including the lens diameter, numerical aperture, and bandwidth of achromatic operation. The achromatic focusing metasurfaces were demonstrated on a dielectric platform of amorphous silicon nanostructures on a quartz substrate. By joint utilization of the spectral degrees of freedom in the lens phase profiles and the geometric degrees of freedom in the meta-units, they created achromatic metalenses that reach the theoretical limitations on chromatic aberration correction (Fig. 6a). With similar design principles, Fan et al. realized a silicon nitride metalens in the visible region with zero effective material dispersion and an effective achromatic refractive index distribution from 430 to 780 nm [75]. They demonstrated a metalens array consisting of $60\times 60$ polarization-insensitive metalenses with nearly diffraction-limited focusing and high efficiency, which can be applied to reconstruct 3D optical scenes in the achromatic integral imaging for the natural white light (Fig. 6b). This metalens array is composed of only a single ultrathin silicon nitride layer that is compatible with on-chip hybrid-COMS integration and the parallel manipulation of optoelectronic information. It is important to mention that a degree of freedom in the design space is sacrificed in favour of the isotropic symmetry requirement on individual meta-units for polarization-independence. This greatly limits the potential to optimize for practical applications.

**Fig. 7:**
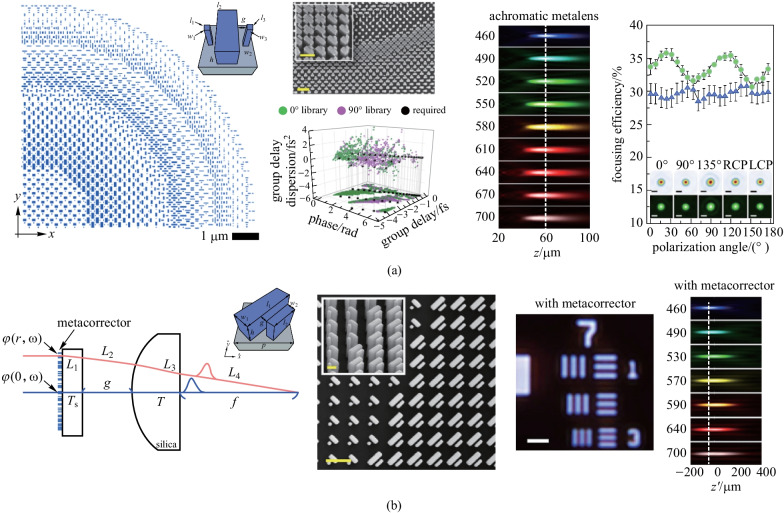
Polarization insensitive broadband metalens with anisotropic unit-cells. **a** A broadband achromatic metalens with a NA of 0.2 over the visible range from 460 to 700 nm while simultaneously maintaining polarization-insensitive and diffraction-limited performances. **b** A metacorrector with a tunable phase and artificial dispersion to correct spherical and chromatic aberrations in a large spherical plano-convex lens. **a** Reprinted with permission from Ref. [76]. Copyright 2019, Chen et al. **b** Reprinted with permission from Ref. [77]. Copyright 2018, American Chemical Society

Whereas the above solutions were based on spatial multiplexing and isotropic symmetry, Capasso and coworkers illustrated a broadband polarization-insensitive achromatic metalens using otherwise anisotropic TiO_2_ nanofins that offered additional control of chromatic dispersion and phase profile [76]. Even though the design principle was associated with geometric phase (PB phase), the polarization dependence was circumvented by limiting the rotation angle of each anisotropic unit to either 0° or 90°. Since each meta-unit was comprised of multiple nanofins, additional geometric degrees of freedom were offered to better engineer the dispersion. With the variable length, width, and in-between gap size of multiple nanofins, these anisotropic nanostructures allowed for a more accurate implementation of the phase and its two lowest order derivatives (group delay and group delay dispersion) with respect to frequency in Eq. (8). Therefore, they achieved a broadband achromatic metalens with a NA of 0.2 over the visible range from 460 to 700 nm while simultaneously maintaining polarization-insensitive and diffraction-limited performances (Fig. 7a). However, all these single-layer polarization-insensitive achromatic metalenses have limited diameters on the order of 100 µm as the consequence of large required group delays. Chen et al. circumvented this limitation and designed a metacorrector by combining a tunable phase and artificial dispersion to correct spherical and chromatic aberrations in a large spherical plano-convex lens (Fig. 7b) [77]. These metacorrectors utilized anisotropic nanofins to maintain an accurate phase profile to correct monochromatic aberration, and artificial dispersion was introduced for tailoring chromatic aberration. With local tailoring of the effective refractive index, a variation in light confinement in sub-wavelength waveguides was achieved for the device tunability. The tandem incorporation of metasurfaces and traditional lenses overcame the challenge of obtaining the large required group delay across the whole metalenses. The broadband achromatic metasurface-refractive device in the visible spectrum range brought tremendous progress in simplification and miniaturization of optical systems.

## Angular dispersion manipulation

**Fig. 8:**
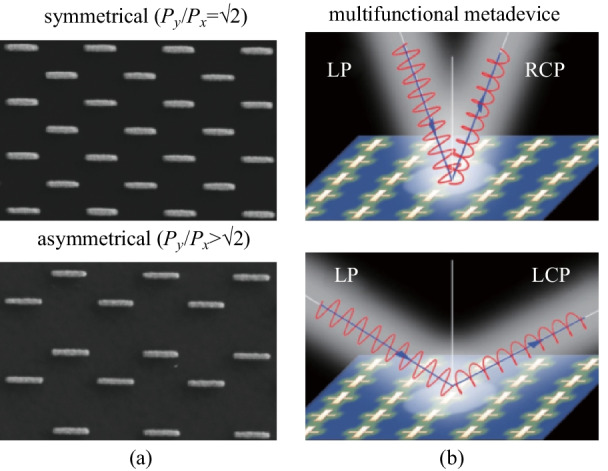
Novel meta-devices to control angular dispersion. **a** SEM images of the angular independent meta-absorber with symmetric and asymmetrical configurations. *P*_*x*_, *P*_*y*_ are the periodicity along *x* and *y* directions, respectively. **b** Schematics of multifunctional metadevice for polarization conversion. Linear incident light can be converted to right-handed (RCP) or left-handed circular polarization (LCP) depending on incident angles. **a** Reprinted with permission from Ref. [78]. Copyright 2020, Zhang et al. **b** Reprinted with permission from Ref. [79]. Copyright 2018, American Physical Society

Metasurfaces have the ability to independently control light incoming from different angles, which is another critical degree of freedom in light focusing. This task is challenging mainly for two reasons: first, a metasurface shows a high angular dispersion which originates from its diffractive nature. On the other hand, the scattering properties of the meta-atoms are generally insensitive to the incident angle. Most of those light-manipulation effects in metasurfaces were only demonstrated under normal-incidence excitation, and the angular dispersions of the devices were mostly unsolved. However, angular dispersion is a crucial issue that must be carefully addressed in various applications such as imaging and solar energy harvesting. Zhou and his coworkers proposed a general strategy to engineer the angular dispersions by carefully controlling both the near-field couplings between meta-atoms and the radiation pattern of a single meta-atom [78]. Specifically, they used a generic lattice configuration with each row shifted a distance with respect to its adjacent row and then considered only the lower-order inter-meta-atom couplings; they thus realized an angular-dispersionless meta-absorber (Fig. 8a). Their approach offered a solution to control the angular dispersion of a metasurface without changing its periodicity or constituent meta-atoms, differently from previous works that have typically been based on enlarging the inter-meta-atom separations [79]. Other than the meta-absorber, Zhou and his coworkers designed a polarization-control metasurface operating under different incident directions (Fig. 8b). In this work, they studied the angular dispersions in periodic metasurfaces that exhibited opposing angular dispersions for the two polarizations. They revealed that the angular dispersions in metasurfaces were determined by plasmonic near-field coupling and provided an additional degree of freedom for design of multifunctional metadevices.

Angular dispersion in metalenses should be specifically discussed for a better understanding of angular response in improving their performances such as imaging quality. As mentioned above, the hyperbolic phase profile (Eq. (5)) can convert the incident wavefront into a spherical one and achieve diffraction-limited focusing without spherical aberration. Nevertheless, such a metalens suffers from other third-order aberrations like coma and astigmatism when illuminated obliquely so its field of view (FOV) is significantly limited. To tackle this problem, several innovative strategies have been proposed in recent years. As will be discussed below, aplanatic metalenses [80], metalens doublets [81–85], aperture-stop-metalenses [86–90], and quadratic phase [91–95] metalenses were adopted to alleviate the aberrations.

### Aplanatic metalens

**Fig. 9:**
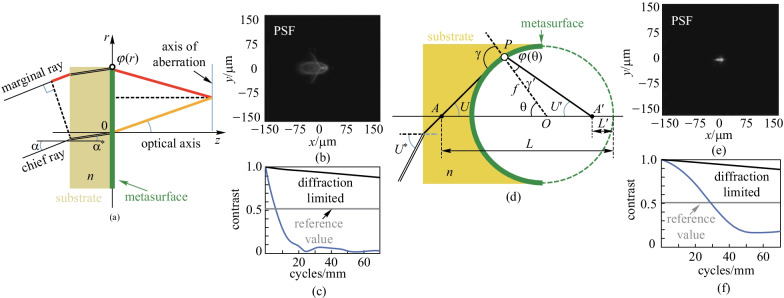
Aplanatic metalens. **a** A flat metalens illuminated by parallel rays incident at angle $\alpha$. The OPD equals the red segment plus the equivalent OP of phase discontinuity $\frac{\lambda }{2\uppi }\phi (r)$, subtracting the yellow segment. **b** Point spread function (PSF) and **c** modulation transfer function (MTF) of flat metalens. **d** Schematics of an aplanatic metalens with metasurface pattern on a spherical substrate. **e** PSF and **f** MTF of aplanatic metalens. The sidelobe here is significantly reduced compared with **b**. And the spatial resolution at minimum contrast 0.5 is enhanced from 8 to 30 cycles/mm. **a**–**f** Reprinted with permission from Ref. [80]. Copyright 2013, The Optical Society

Aieta and coworker pointed out that the optical path difference (OPD) between marginal rays and chief rays (Fig. 9a) that causes the aberrations in flat metalenses could be expanded to polynomials as [80]

9 $$\begin{array}{c}{\text{OPD}}=-\frac{1}{4f}{r}^{2}{\alpha }^{2}-\frac{5}{4f}{r}^{2}{\alpha }^{2}-\frac{1}{4{f}^{2}}{r}^{3}\alpha +{\text{higher order terms}},\end{array}$$

where *α* is the illumination angle and *r* is the radial coordinate. The third term of the polynomials corresponds to coma which is regarded as the most problematic aberration due to the asymmetric distortion it induced in the image. It was pointed out that the spherical aberration and coma can be corrected simultaneously if the Abbe sine condition can be satisfied. Referring to the diagram in Fig. 9d, the Abbe sine condition is equivalent to $\overline{AP }/\overline{A{^{\prime}}P}=\text{sin}U{^{\prime}}/\text{sin}U=\text{constant}$, which implies that the trace of point *P* is an Apollonius circle. On these grounds, they calculated the phase gradient of the spherical metalens required, which reads

10 $$\frac{\text{d}\varphi }{\text{d}\theta }=-n\frac{2\uppi }{\lambda }\text{sin}\theta .$$

To verify the aberration corrections of the phase gradient above, they compared the image quality of the flat metalens with hyperbolic phase to that of the aplanatic metalens with the same radius $\rho =1\text{ mm}$ and NA = 0.5 under the same illumination angle ($\alpha =10^\circ$). Figure 9b, e demonstrate the measured point spread function (PSF) of flat and aplanatic metalenses. The sidelobe is sharply reduced after alternating the flat metasurfaces with aplanatic ones. Figure 9 c and f show the modulation transfer functions (MTF) of the two metalenses, which indicates the normalized contrast transferred by the lens from object to image and characterizes the spatial resolution of the metalens. With the reference minimum contrast fixed at 0.5 (dashed line in Fig. 9c, f), the spatial resolution can be improved from 8 cycles/mm (flat) to 30 cycles/mm (aplanatic) approximately.

### Metalens doublet

**Fig. 10:**
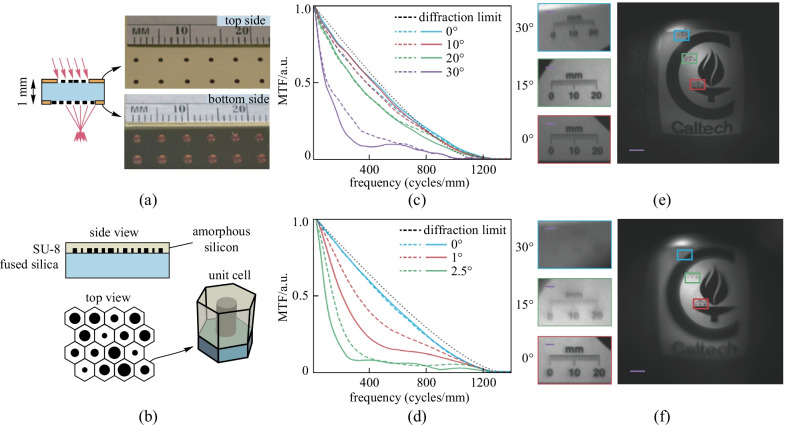
Cascaded metalenses in the near infrared. **a** Schematic of the aberration-free metalens doublet focusing off-axis light. **b** Illustration of the dielectric metasurface used to implement the metalens. The metasurface array is composed of amorphous silicon posts with variant diameters and SU-8 polymer on top in hexagonal arrangement. The MTF of **c** polynomials doublet and **d** hyperbolic singlet metalens. The focal length and aperture diameter of both lens is set as the same. Image taken by **e** the doublet and **f** the singlet. **a**–**f** Reprinted with permission from Ref. [81]. Copyright 2016, Arbabi et al.

Although the aplanatic strategy mentioned above can broaden the FOV, this method poses a non-trivial challenge for fabrication, inevitably increases the cost, and sacrifices the flatness of metasurfaces. To obtain wide-angle aberration-free imaging using a flat metalens, the cascade strategy was proposed, namely the metalens doublet. As for conventional bulk optical system design principles, the metalens doublet is comprised of a focusing metalens, with a correcting metalens positioned in front to reduce the coma and astigmatism. Based on this approach, Arbabi et al. demonstrated a metalens doublet (Fig. 10a, b) which has a FOV larger than 60° and exhibits near-diffraction-limited imaging at a wavelength of 850 nm [81]. The focusing efficiency of this metalens ranges from 45% to 70% depending on the incident angle.

Different from the quadratic phase, the metalens proposed here adopted another wisely designed phase profile by using a ray-tracing technique. In this design, the phase profile is defined as an even order polynomial of the radial coordinate $\rho$:

11 $$\begin{array}{c}\varphi \left(x,y\right)=M\sum_{i=1}^{n}{a}_{n}{\left(\frac{\rho }{R}\right)}^{2i},\end{array}$$

where *M* is the diffraction order, *R* is the radius of the metalens, $\rho =\sqrt{{x}^{2}+{y}^{2}}$ is the distance from the point (*x*, *y*) to the center of the metalens. The coefficients ${a}_{n}$ are optimized by minimizing the focal spot sizes at incident angles up to 30°.

The focusing and imaging performances of this metalens doublet are compared with a hyperbolic singlet metalens in Fig. 10c and d, which show the MTF of the doublet and the singlet, respectively. The spatial resolution of the doublets remains near D.L. at large angles while the MTF of the singlet deviates from the D.L. rapidly under slightly oblique illumination. Correspondingly, Fig. 10e and f present the images of both metalenses, indicating that the blurring on edges is much more severe in the singlet image.

**Fig. 11:**
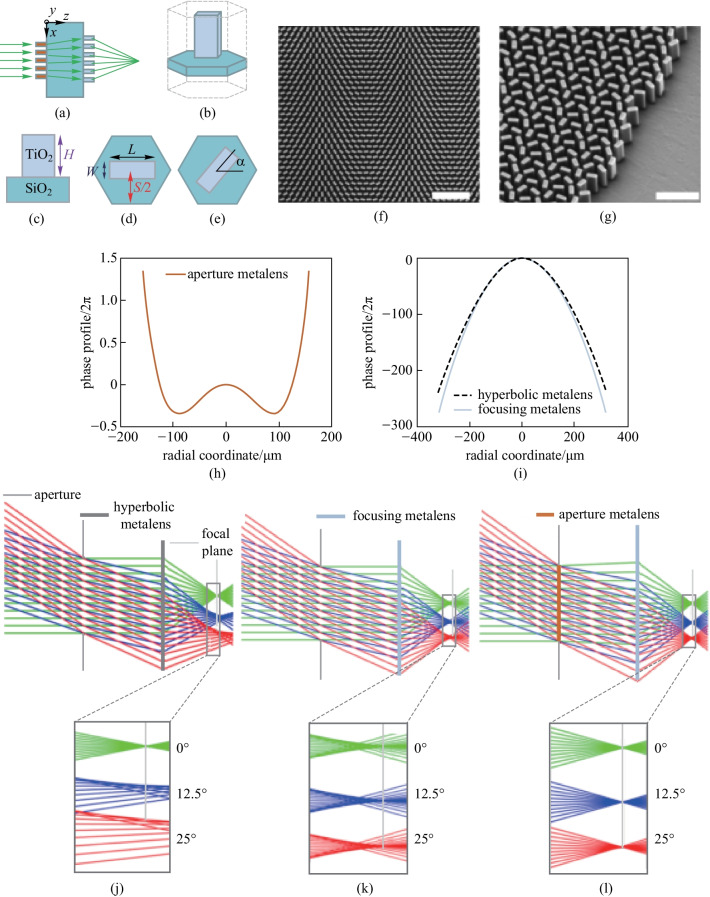
Metalens doublet in the visible: schematic illustration, SEM image and phase profile of the metasurface doublet. **a** Metalens doublet is comprised of two metasurfaces integrated on both sides of a SiO_2_ substrate. **b**–**e** Geometrical parameters of the TiO_2_ nanofins; **c**–**e** side and top views of the hexagonal unit cell with constant periodic length *S*, nanofin height *H*, nanofin length *L*, width *W*, and variant rotation angle $\alpha$. **f** Top-side view SEM micrograph of the focusing metalens. **g** Side view SEM micrograph at the edge of the sample. **h** Phase plot of aperture metalens. **i** Comparison of phase plots of hyperbolic metalens and that of focusing metalens designed based on Eq. (10). **j**–**l** Ray diagrams to depict the principle of aberration correction. **j** Ray diagram of hyperbolic metalens which shows large aberration at oblique illuminance. **k** Ray diagram of metalens with phase profile designed according to light blue curve in Fig. 9g, which shows positive and negative spherical aberration. **l** Ray diagrams of the metalens doublet showing diffraction-limited focusing at all angles. **a**–**l** Reprinted with permission from Ref. [82]. Copyright 2017, American Chemical Society

Likewise, Groever et al. also developed a cascaded metalens in the visible region [82]. In their design, an aperture metalens was placed before the focusing lens, acting as a correcting metalens. The name aperture metalens was designated because the metalens plays the same role as the aperture in a Chevalier Landscape Lens, the first camera lens widely used after the invention of photography [96]. The metalens doublet exhibits a FOV as large as 50° at 532 nm wavelength which enables diffraction-limited imaging. This metalens is comprised of TiO_2_ nanofins array in hexagonal arrangement (Fig. 11a–e) with different rotation angles to impart a designed phase profile $\phi \left(x,y\right)$(Fig. 11f and g). As PB phase is used here, the metalens designed is sensitive to the polarization of incident light. The phase of the aperture metalens is chosen as even order polynomials:

12 $$\begin{array}{c}{\phi }_{\text{AL}}\left(x,y\right)={\sum }_{i=1}^{5}{{a}_{i}\left(\frac{\rho }{2{R}_{\text{AL}}}\right)}^{2i},\end{array}$$

and the phase of focusing lens is chosen as the summation of hyperbolic and polynomials profile:

13 $$\begin{array}{c}{\phi }_{\text{FL}}\left(x,y\right)=-\frac{2\uppi }{\lambda }\left(\sqrt{{x}^{2}+{y}^{2}+{f}^{2}}-f\right)+{\sum }_{i=1}^{5}{{b}_{i}\left(\frac{\rho }{2{R}_{\text{FL}}}\right)}^{2i},\end{array}$$

where ${{R}}_{\text{AL}}\text{ and } {R}_{\text{FL}}$ are the radii of the aperture metalens and focusing metalens, respectively. And the phase plots of aperture and focusing metalens based on Eqs. (12) and (13) are shown in Fig. 11h and i, respectively.

According to Fig. 11i, the phase gradient of the focusing metalens is slightly larger than the hyperbolic one and consequently the marginal rays will be bent more strongly toward the optical axis than the chief rays. Therefore, the focusing metalens (without aperture lens, Fig. 11k) has more uniform ray diagrams at different incident angles than hyperbolic metalens (Fig. 11j). This provides the possibility to correct the aberration with another metasurface. The phase plot of aperture metalens (Fig. 11h) is reminiscent of the Schmidt plate, an optical element that can converge the chief rays while diverging the marginal rays. With the presence of aperture metalens, the positive and negative spherical aberrations presented in Fig. 11k can be perfectly compensated to exhibit diffraction-limited imaging along the focal plane at incident angles up to 25° (Fig. 11l).

### Metalens singlet with aperture stop

Although the cascaded metalens provides an extra degree of freedom to reduce the off-axis aberration, introducing another metasurface increases the cost and poses difficulties for integration. In recent works, it has been demonstrated that by adding an aperture stop some distance in front of the metalens, in addition to spatially modified phase profile, coma and other third-order aberrations can be corrected and one can achieve wide FOV with a single metasurface.

**Fig. 12:**
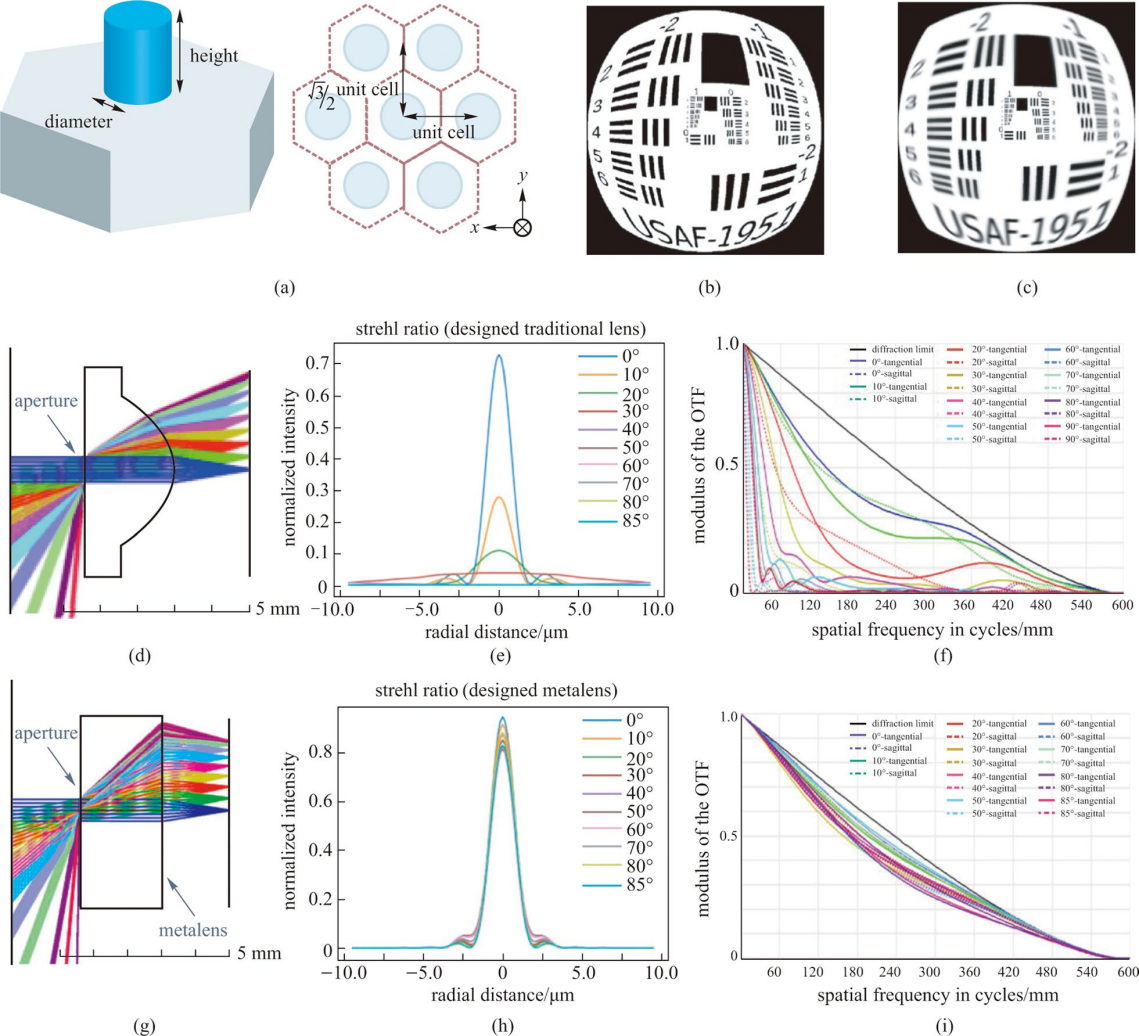
Wide-angle metalens with aperture stop. **a** Schematic of the hexagon unit cell composed of SiO_2_ nanopost placed on GaN substrate with fixed height 600 nm. **b** Simulated images of USAF-1951 test chart with traditional lens and **c** wide-angle metalens. **d** Traditional lens layout. **e** Traditional lens Strehl ratio. **f** Traditional lens MTF. **g** Metalens layout. **h** Metalens Strehl ratio. **i** Metalens MTF. The NA of the optical system in both the designs is set to 0.18, and the cutoff frequency is approximately 600 in cycle per mm. **a**–**i** Reprinted with permission from Ref. [86]. Copyright 2020, Fan et al.

Following this method, Fan et al. [86] proposed a wide-angle metalens singlet using SiO_2_ nanoposts patterned on a GaN substrate (Fig. 12a). The metalens provides a diffraction-limited FOV of over 170° with a NA of 0.25 at 532 nm. Figure 12b and c show the simulated images of the USAF-1951 test chart created with a traditional lens and a wide FOV metalens. The results indicate that the metalens design can maintain high imaging quality at large angles and can correct the coma. As in the ray diagram shown in Fig. 12g, the presence of aperture ensures that light rays from different incident angles can be directed onto corresponding parts of the metasurface and provides the possibility to design a phase profile suitable for focus of light from all directions. The phase adopted here is also in the form of even order polynomial as Eq. (13), determined by ray-tracing technique.

To analyze the designed metalens, the Strehl ratio and MTF were calculated for the single traditional lens and the designed metalens at the wavelength of 532 nm. The Strehl Ratio is defined as the ratio of the peak aberrated image intensity from a point source with the maximum attainable intensity using an ideal optical system limited only by diffraction. The Strehl and MTF of the single traditional lens and designed metalens are shown in Fig. 12e, f, h, i, respectively. The Strehl ratio of the designed metalens is larger than 0.8 (the industrial standard threshold to be classified as “diffraction-limited”) even at an angle as large as 85° and the MTF is also near diffraction limits at each angle in the metalens design.

### Quadratic phase profile

As discussed in the last two sections, the cascaded metalens and aperture-stop-integrated metalens can significantly broaden FOV while maintaining diffraction-limited performance. However, both methods have to include aperture stop and this limits the effective aperture size one can achieve. Moreover, in the latter case only a limited fraction of the metasurface is used and the light throughput is reduced. To realize angular dispersion free focusing on single layer metalens without stop, a phase profile with quadratic form is proposed by Pu et al. [93] by reducing the requirement on diffraction limit:

14 $$\begin{array}{c}\varphi \left(r\right)=k\frac{{ r}^{2}}{2f}+kx\text{sin}\theta =\frac{k}{2f}\left({\left(x+f\text{sin}\theta \right)}^{2}+{y}^{2}\right)-\frac{fk{\text{sin}}^{2}\theta }{2}.\end{array}$$

**Fig.13:**
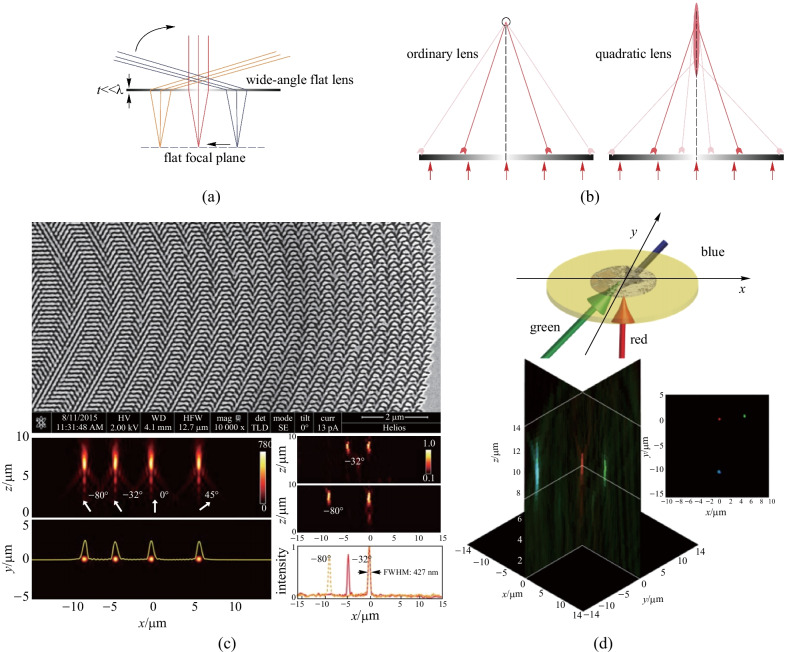
Proof-of-concept quadratic phase metalens. **a** Ray diagram of wide-angle flat lens illuminated by oblique rays. Red, yellow and blue rays are corresponding to different incident angles. The lens transforms the difference in incident angles into traverse shifts of focuses on the focal plane. **b** Ray diagram of an ordinary lens and a quadratic flat lens at normal illumination. Spherical aberration is introduced in the quadratic lens. **c** Top: SEM of the fabricated metalens with elliptical aperture arrays on a gold film. Bottom left: simulated results of light intensity distributions on *xz* (*y* = 0) and *xy* (*z* = 7.5 μm) plane at 632.8 nm with $0^\circ ,-32^\circ ,-80^\circ ,\text{and } 45^\circ$ incident angles. Bottom right: experimental measurement of light intensity distribution on *xz* plane at $\theta =0^\circ ,-32^\circ$ and $\theta =0^\circ ,-80^\circ$. The FWHM is about 427 nm. **d** Top: schematic of measurement set up to demonstrate the multiwavelength behavior. Bottom: intensity distribution in a common focal plane shows clear spots for three wavelengths. **a**–**d** Reprinted with permission from Ref. [93]. Copyright 2017, The Optical Society

This profile is a paraxial approximation of the hyperbolic phase. On the periphery of the metalens, the radial wavenumber ${k}_{r}$ (defined as $\partial \varphi /\partial r$) is larger so the rays are bent more severely and this introduces spherical aberration even at normal incidence (Fig. 13b). At normal incidence, the light passing through regions where *r* > *f* will become evanescent and therefore does not contribute to the focal spot. This characteristic of the quadratic phase can effectively play the role of a physical aperture stop. As the last term in Eq. (14) is independent of *r* and thus can be neglected, there is only a traverse shift of $f\text{sin}\theta$ on the focal plane with respect to the normal incidence. Thus, at oblique illumination, the evanescent zone will shift horizontally. As a result, the phase gradient introduced by $kx\text{sin}\theta$ converts rotation symmetry to translation symmetry (Fig. 13a). To some extent, the quadratic phase profile is a trade-off between FOV and spatial resolution. However, the image quality of such a metalens is sufficient in most applications.

Using the phase they proposed, Pu et al. demonstrated a proof-of-concept metalens that can correct coma and astigmatism at angles as large as 80°. The metalens was fabricated by defining elliptical nano-aperture on an ultrathin gold film (Fig. 13c top). The phase profile is imparted by rotating the apertures in a hexagonal lattice. Simulated intensity distributions on the crosssection of *xz* (*y* = 0) and *xy* (*z* = 7.5 μm) plane are shown in Fig. 13c bottom left. As shown in the simulated result, at *λ* = 632.8 nm the focal spots of different incident angles are virtually the same except that there are translational shifts corresponding to *f*sin $\theta$. The measurement results shown in Fig. 13c bottom right demonstrate the focusing behavior of the sample at normal and − 32°, − 80° incidence, which agree well with the simulation results.

As mentioned, the large depth-of-field (DOF) of the quadratic phase causes undesirable spherical aberration. However, it can be utilized to solve the achromatic aberration to some extent. Specifically, although the centroids of the focused beam would shift to larger *z* when the incident wavelength increases, the focal spot can be observed clearly in a common section if the shifts induced by chromatic dispersion can be tolerated by the large DOF (Fig. 13d).

**Fig. 14:**
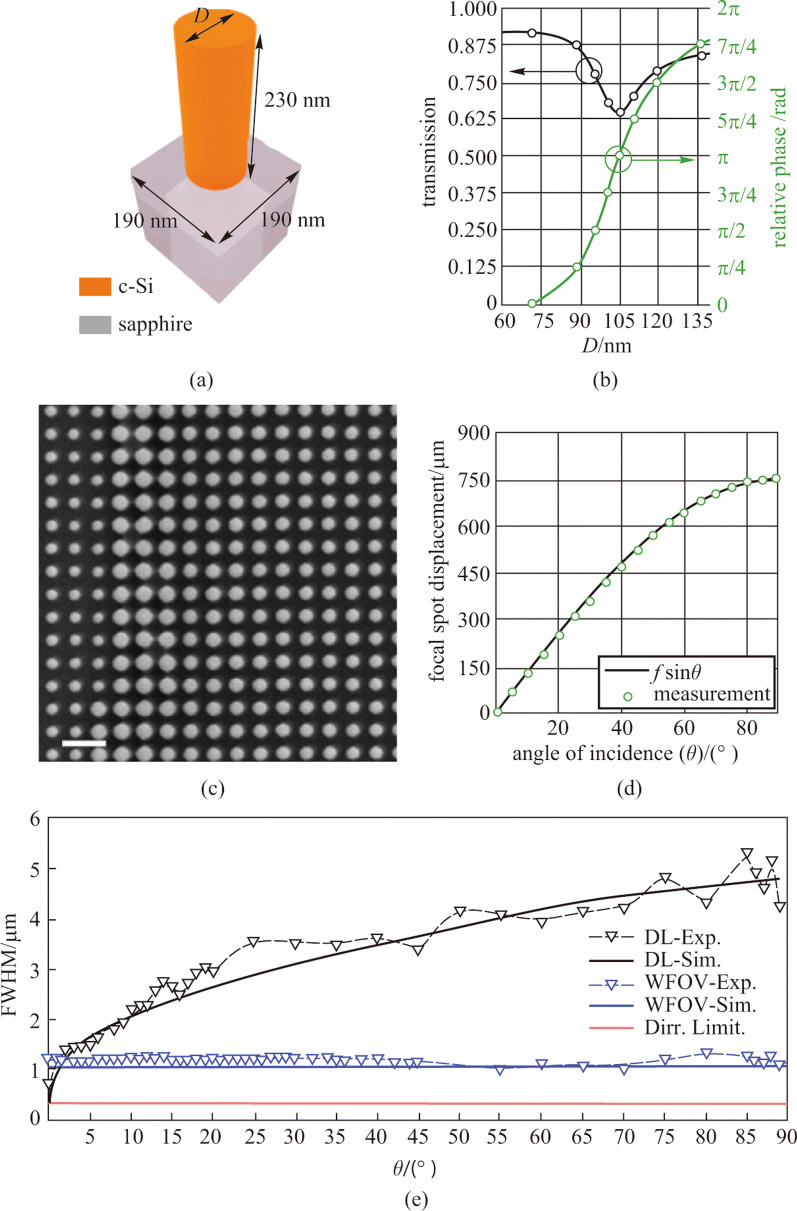
Quadratic phase metalens with arbitrarily wide FOV. **a** Schematic of the c-Si nanopost unit cell with fixed period *a* = 190 nm and height *h* = 230 nm. **b** Transmission and phase map. *D* refers to the diameters of c-Si nanoposts and the cycle marks represent the eight phase levels used to discretize the phase profile. **c** SEM micrograph of the c-Si nanopost array (top view). **d** Measured displacement of the focal spot as a function of incident angels. **e** Measured and simulated FWHM versus incident angles curves of hyperbolic (referred to as D.L. in the graph) and quadratic (referred to as WFOV) lens. **a**–**e** Reprinted with permission from Ref. [92]. Copyright 2020, American Chemical Society

Based on the quadratic phase profile, A. Martins and coworkers demonstrated a single-layer flat metalens composed of c-Si nanoposts patterned on a sapphire substrate which exhibits arbitrarily wide FOV (near 180°) at a wavelength of 532 nm [92]. The full width at half maximum (FWHM) of such a metalens remains at 2${\lambda }_{0}$ for FOV > 170°, which is comparable to bulk optics lens. The meta-atoms are arranged in square unit cell (Fig. 14a) with constant period and the desired phase profile is realized by varying the diameters of each c-Si nanopost. Figure 14b shows the transmission and phase as a function of the diameter calculated by rigorous coupled wave analysis (RCWA).

The focal displacement at different incident angles is presented in Fig. 14d and it indicates that the displacement is predictable, which makes it possible to be corrected by postprocessing. Martins et al. further compared the wide angle performance of a quadratic metalens with that of a hyperbolic metalens (referred to as D.L. in Fig. 14e) [92]. It shows in Fig. 14e that the FWHM of the hyperbolic lens increases rapidly with deviation from normal incidence although it can achieve D.L. resolution at 0°. On the contrary, the FWHM of quadratic lens maintains to ~ 2${\lambda }_{0}$ at arbitrary incident angles. However, it cannot be neglected that the focusing efficiency of such wide FOV metalens is only 3.5% at normal incidence, which is quite low comparing with hyperbolic lens (23% at normal incidence). Martins et al. pointed out that the low efficiency of the wide-angle lens is a consequence of the combination of spherical aberration and effective numerical aperture [92]. The NA was reduced as the effective area of the metasurface that can concentrate light was smaller in this case. And the spherical aberration introduced by quadratic phase makes chief contribution to the low efficiency here.

To summarize, some key performance merits of the aforementioned wide-angle metalenses are listed in Table 1. Historically, use of the aplanatic metalens is the earliest strategy proposed to solve the angular dispersion. However, this method loses the most important advantage of metasurfaces, namely, flatness, and poses severe challenges in fabrication. These limit its applications and few further works have been reported subsequently. The cascaded metalens can generally broaden the diffraction-limited FOV to about 60° with relatively high efficiency and large numerical aperture. Yet these metalenses cannot achieve panoramic imaging (180° FOV) and increase cost and fabrication complexity. As a paraxial approximation to the hyperbolic phase, quadratic phase shows remarkable ability in correcting angular dispersion. Nevertheless, the spherical aberration induced and the consequential super-low efficiency limit the practicability of this approach. In contrast, the focusing efficiency of the aperture-stop metalens is at an acceptable level even though its NA is largely limited by the stop. Moreover, the aperture-stop metalens can achieve near panoramic diffraction-limited FOV and thus exhibits the most balanced performance merits.

**Table 1:** Summary of key performance merits for wide FOV metalens

Reference	Method	FOV/(°)	Efficiency/%	Layers	Wavelength/nm	NA
Aieta et al. [80]	Aplanatic	~ 20	–	1	1550	0.5
Arbabi et al. [81]	Doublet	56 (DL)	45–70	2	850	0.49
Groever et al. [82]	Doublet	50 (DL)	30–50	2	470–660	0.44
Fan et al. [86]	Aperture Stop	> 170 (DL)	45–82	1	532	0.25
Martin et al. [92]	Quadratic	180	< 3.5	1	532	0.8

DL: Diffraction limits

## Applications and outlook

In light of the advantages such as compactness, light weight, miniaturization compatibility and low cost, metasurfaces exhibit exciting potentials to replace their bulky conventional counterparts in various applications. In this section, we elaborate the applications of metalenses including microscopic imaging [97–100], spectroscopy [101–107], full-color routing [108–110], chiral sensing [111–113], and solar energy harvesting [114–116].

### Imaging

**Fig. 15:**
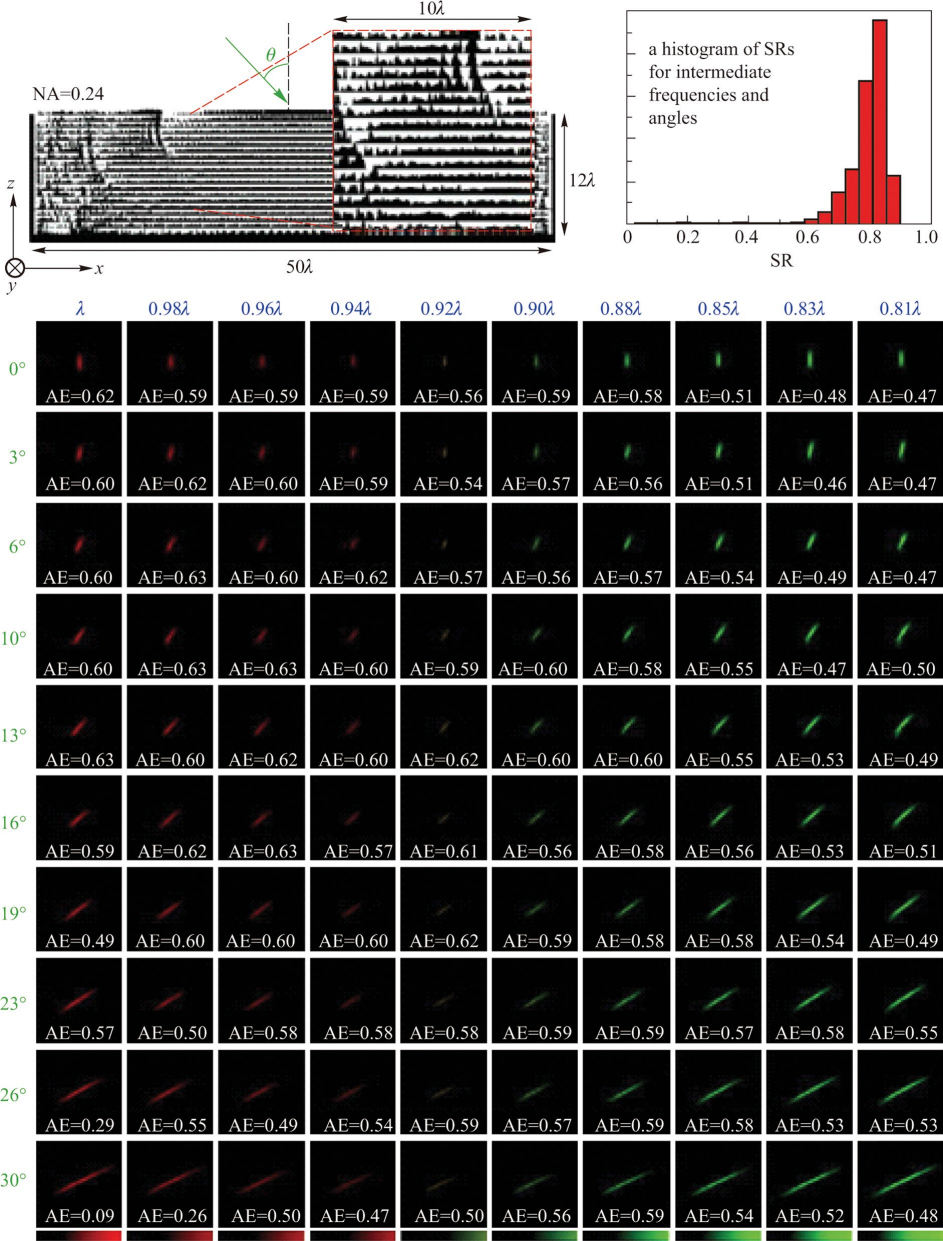
Inverse designed single-piece multilayer metalens that simultaneously corrects chromatic and angular aberrations. Top-left is the schematic of the metalens consisting of 20 layers of 3D-printable polymers with NA = 0.24. Top-right inset shows the distribution of Strehl ratio (SR) of intermediate frequencies and angles within the designated bandwidth and FOV. Most SRs remain higher than 0.7 and the mean value is larger than 0.75. The bottom shows the cross-section light field distributions and AEs of the designed wavelengths and angles (*N* = 10 × 10 = 100). The average of AEs is as high as 55%. Reprinted with the permission from Ref. [61]. Copyright 2021, AIP Publishing

As mentioned in previous sections, metalenses exhibits high quality imaging abilities such as achromatic focusing with high focusing efficiency and spatial resolution near diffraction limits in addition to wide FOV. However, it’s noteworthy that the aforementioned works are dealing with chromatic and angular aberration separately. How to realize the combination of achromatism and wide FOV in one metalens is an intriguing challenge. In Ref. [61], Lin et al. designed a single-piece metalens using topology optimization that exhibits 23% bandwidth and an over 60° FOV without chromatic and angular aberrations. Moreover, their computational results showed that the metalens can achieve diffraction-limited performance and an average focusing efficiency of over 50% at the designed frequencies and incident angles. To realize this, they set the minimum of Strehl ratios for multiple frequencies and multiple angles as the objective function, and then employed a gradient-based optimization method to search for maximum value of the objective function in the design space taking into account the dielectric permittivity at spatial points as design variables. As shown in Fig. 15, they presented a metalens comprised of 20 layers of 3D-printable polymer with a footprint thickness of 12$\uplambda$ which can realize aberration-free focusing at 10 frequencies and 10 angles within 23% bandwidth and over 60° angular range, respectively. The simulated results showed the Strehl ratios (SRs) for designed frequencies and angles are approximately 0.89 and remained higher than 0.75 in between. In addition to the Strehl ratio, the average absolute focusing efficiency (AE) was as high as 55%, which is better than any previous achromatic metalens.

**Fig. 16:**
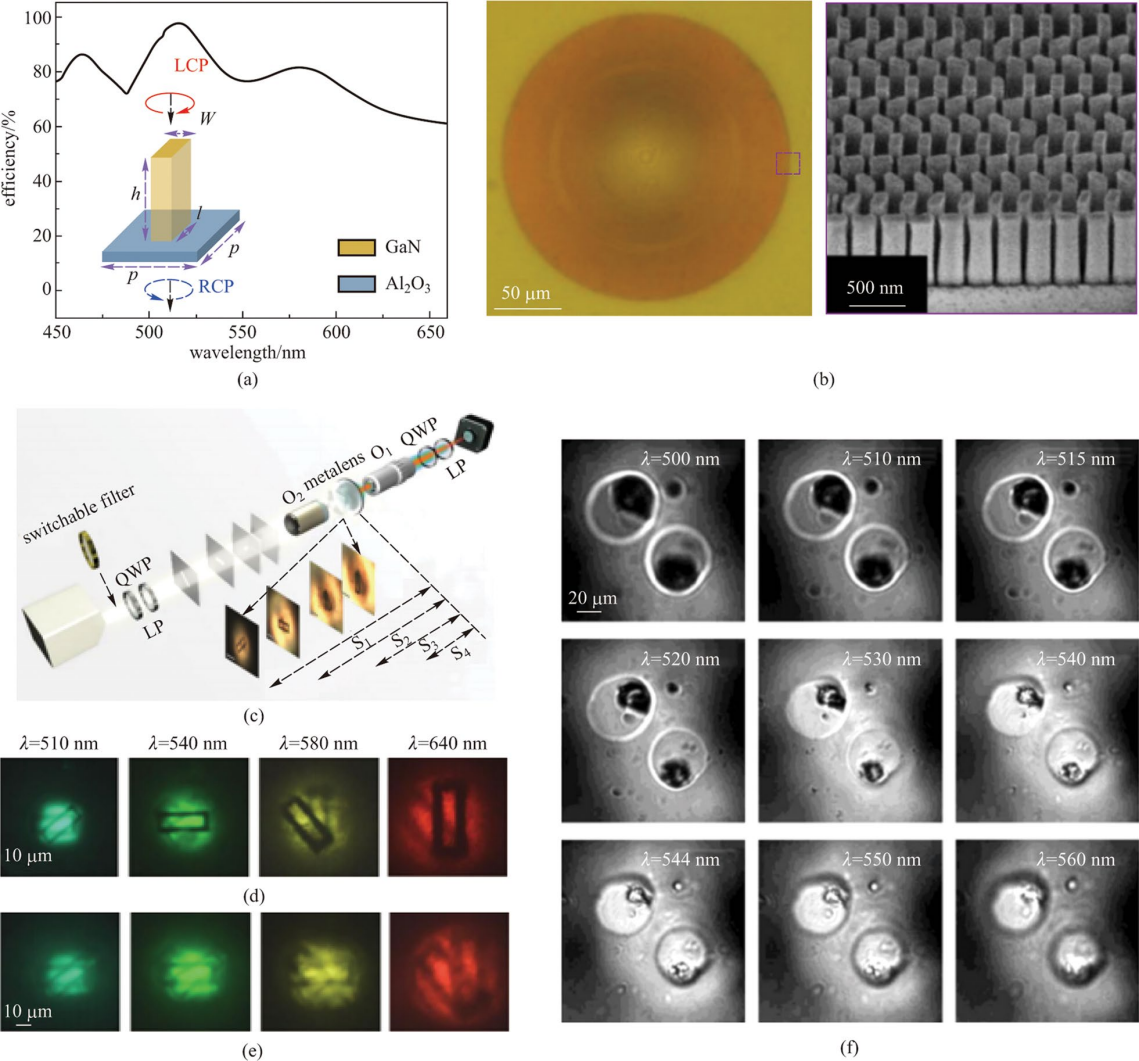
Spectral tomographic imaging system based on aplanatic metalens. **a** Calculated focusing efficiency of the unit cell over working wavelength. The inset is a schematic of the unit cell composed of GaN nanopost placed on sapphire. **b** Optical (left) and SEM (right) images of the fabricated metalens. **c** Schematic of the imaging setup. The inset shows four images obtained by an objective O_2_ acting as objects to verify the tomographic imaging of the metalens. Images captured by **d** aplanatic and **e** normal metalens through the objective O_1_ and CCD at different wavelengths are shown. **f** Microscopic tomography of frog egg cells by aplanatic metalens at different incident wavelengths. **a**–**f** Reprinted with permission from Ref. [99]. Copyright 2019, Chen et al.

Although chromatic dispersion is usually a negative phenomenon in imaging systems, it can be carefully employed in some other applications to achieve desired functions. Utilizing large chromatic dispersion of an aplanatic metalens, Chen et al. proposed a novel spectral tomographic imaging system without complex mechanical scanning components in a non-motion design [99]. They implemented the aplanatic phase profile by imparting geometry phases to the GaN nanopost array and demonstrated a high-resolution (775 nm) metalens in visible wavelength (450–660 nm) with NA = 0.78 (Fig. 16a, b). The large chromatic dispersion of the metalens made the focal length tunable and by simply switching the incident wavelength they could realize non-motion optical zooming-in and DOF scanning (Fig. 16c–e). They further imaged a biological specimen of frog egg cells via tomography by the metalens. Figure 16f shows a group of images obtained by the metalens with wavelength increased from 500 to 560 nm. The results indicate that the membrane and the nucleus are at different depths of field.

### Spectroscopy

Spectroscopy is another typical application of metalenses utilizing chromatic dispersion. To enhance the resolution, a conventional spectrometer that depends on the dynamic phase has to increase the distance between the grating and the detector, making it difficult to miniaturize and compact. Khorasaninejad and coworkers proposed [101] an off-axis metalens that simultaneously focused and dispersed the incident light at different wavelengths to achieve high spectral resolution in a compact configuration. Silicon nanofins were used as unit-cells and the phase profile was implemented by rotating the orientation of the meta-atoms. Hyper-resolution of the incident wavelengths could be achieved by utilizing the dispersive characteristic of the metalens.

**Fig. 17:**
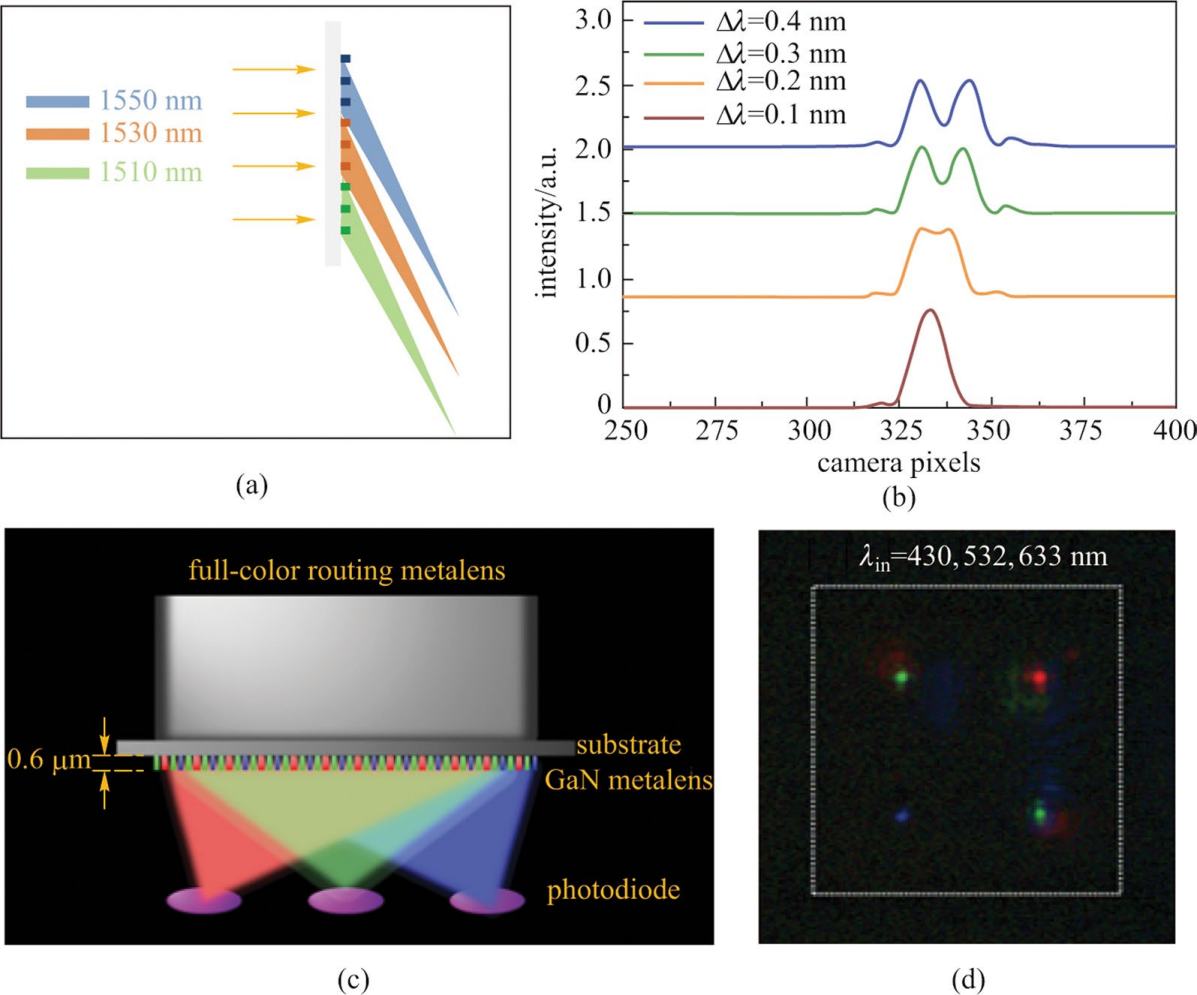
Spectroscopy and full-color routing applications of metalens. **a** Schematic of the off-axis super-dispersive metalens. Several metalenses with different working wavelengths are stitched together to extend the bandwidth while maintaining high resolution. **b** Spectrum at focusing angle of 80°. The spectral resolution is as high as 0.2 nm. **c** Schematic of GaN metalens integrated with complementary metal–oxide–semiconductor (CMOS) combining light convergence and color filtering functionalities. **d** Measured field intensity on the focal plane (cross-section of *x–y* plane) with three different colors illumination: blue, green, and red. **a**, **b** Reprinted with permission from Ref. [101]. Copyright 2016, American Chemical Society. **c**, **d** Reprinted with permission from Ref. [109]. Copyright 2017, American Chemical Society

Khorasaninejad et al. pointed out that the dispersion increases as the focusing angle increases [101]. When focusing at an angle of 80°, the angular dispersion of the metalens is as high as 0.27 nm/mrad which surpasses the conventional spectrometer. Such precision makes it capable of resolving spectral differences in the order of 200 pm. Meanwhile, the focusing efficiency maintains as high as 90% over the working wavelengths of 1.1–1.6 μm. What’s more, the high precision can be preserved to a wider bandwidth by stitching metalens with different working wavelength ranges together (Fig. 17a). In light of its high resolution, flatness, and miniature size, the off-axis metalens-based spectrometer has exhibited potentials in applications like compact and portable optical devices.

### Full-color routing

As another example of utilizing the chromatic dispersion, Chen et al. demonstrated a GaN-based metalens to direct three primary color light into arbitrary position in free space with extremely high efficiency (Fig. 17c) [109]. To separate different wavelengths of light into different positions, four out-of-plane focusing metalenses based on PB phase were integrated into one sample. Unlike the super-dispersive off-axis spectrometer, the full-color router metalens can focus three primary color lights, namely green (G, 430 nm), blue (B, 532 nm) and red (R, 633 nm) into arbitrary spatial position individually (Fig. 17d). Compared with conventional complementary metal–oxide–semiconductor (CMOS) image sensors (CIS), the multiplex color router can function as an integration of micro-lens and color filter, giving rise to its extremely high efficiency of 87%, 91.6% and 50.6% for $\lambda$ = 430, 532, and 633 nm, respectively. Besides color routing, with its low cost, semiconductor fabrication compatibility and high efficiency, the GaN out-of-plane metalens exhibits prospects in applications such as miniature CIS chips, flat optoelectronic circuits and high-resolution lithography.

### Chiral imaging

**Fig. 18:**
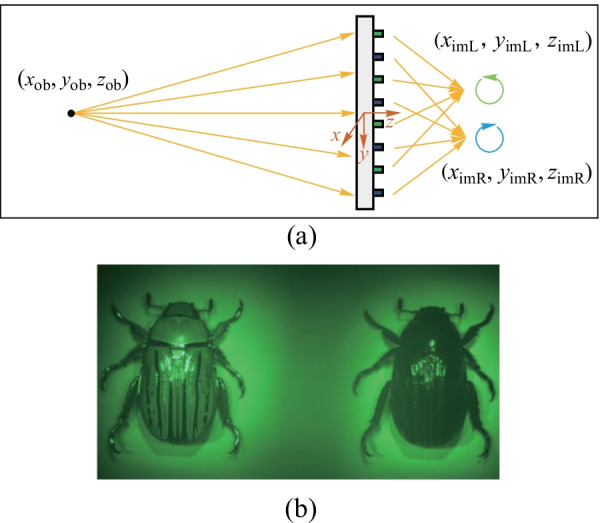
Chiral imaging application of metalens. **a** Schematic diagram illustrating the principle of chiral imaging metalenses. Linear polarized (combination of LHC and RHC) light emitted from an object at coordinates $({x}_{\text{ob}},{y}_{\text{ob}},{z}_{\text{ob}})$ are focused into separate focuses $({x}_{\text{imL}},{y}_{\text{imL}},{z}_{\text{imL}})$ and $({x}_{\text{imR}},{y}_{\text{imR}},{z}_{\text{imR}})$. The nanofins colored blue impart the required phase profile to focus RHC light while the green color ones impart the phase required to focus LHC light. **b** Images of a beetle formed by a chiral imaging metalens under 532 nm LED illumination. The left and right images are formed by focusing the LHC light and the RHC light, respectively. **a** and **b** Reprinted with permission from Ref. [112]. Copyright 2016, ACS Publications

Although our eyes can partially perceive spectral information of light through colors, we are basically blind to the polarization property of incident light. Thus, polarization imaging systems have been invented to utilize the information contained in the polarization property of light and this is significant in chemical and biological specimen analysis due to the intrinsic handedness of certain compounds such as amino acids and glucose. Conventional polarization imaging systems are composed of multiple optical components such as polarizers and waveplates, making them complicated and bulky and meanwhile reducing image quality. Combining two interlaced arrays of TiO_2_ nanofins to a single substrate, Khorasaninejad et al. demonstrated [112] a PB-phase-based metalens that can focus LHC and RHC lights into different positions on the same focal plane (Fig. 18a). What’s more, as the coordinates of focal spots are dependent on wavelength, and due to its dispersive design, the metalens can simultaneously realize multi-spectral resolution. Two images captured by the metalens with opposite chirality of a biological specimen under the same FOV are shown in Fig. 18b. The result shows that the chiroptical property of light can be probed using a single flat metalens across the visible spectrum without additional optical components, paving the way for miniaturization of such devices.

### Solar energy harvesting

**Fig. 19:**
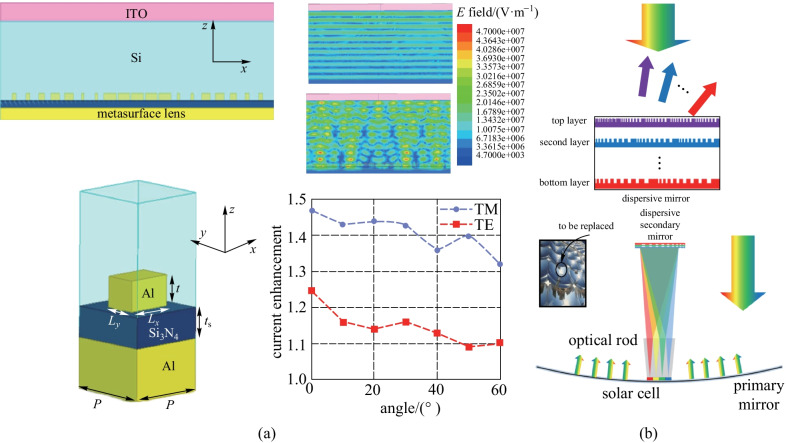
Applications in solar energy harvesting. **a** Metasurface lens integrated into a silicon solar cell to enhance light absorption by trapping light into the active area. The simulated result shows field enhancement at 550 nm incident wave which is TE polarized. The short circuit current exhibits improvement at angles up to $60^\circ$. **b** Multi-layer dielectric high-index-contrast gratings (HCG). Normally incident lights are directed to different angles depending on wavelengths. By replacing the secondary mirror, the HCG dispersive mirror can act as both sunlight concentrator and spectral splitter. **a** Reprinted with permission from Ref. [116]. © The Optical Society. **b** Reprinted with permission from Ref. [115]. Copyright 2014, Springer-Verlag Berlin Heidelberg

Concentrating sunlight is another significant application of metasurfaces. In Ref. [116], Shameli and Yousefi proposed a method to enhance energy absorption by using metasurfaces integrated on silicon solar cells. The aluminum nano-brick was used as a resonator to impart phase continuity required to trap light in the active area of solar cells (Fig. 19a). The numerical results show that the average light field intensity can be enhanced by the resonators patterned on the substrate. As the phase was implemented by tuning the width of the nano-brick, this metasurface can work under both TE and TM polarization. However, it should be noted that the absorption enhancement peaks are discrete on the spectrum. At enhancement peaks, the simulation results indicated that the short circuit current can be increased by factors of 1.22 and 1.15 for TM and TE polarization, respectively. To avoid the ohmic loss induced by the metallic meta-atoms, Shameli and Yousefi also illustrated a dielectric metasurface lens by replacing the aluminum nano-brick with an ITO nano-brick. Numerical results show that the dielectric metalens can increase short circuit current for both TM and TE polarization by factors of 1.47 and 1.25, respectively. Moreover, their simulation demonstrated that the short circuit current shows improvement for large incident angles up to $60^\circ$.

Improving the utilization of the whole sunlight spectrum is crucial in photovoltaic power systems. The parallel configuration of integrated multiple junctions has been developed to realize spectrum splitting absorption because a single p-n junction can only convert photons with higher energy than its bandgap and only part of the photon energy can be converted into electricity. However, most parallel spectral splitting is realized by optical filters and geometrical optics, which induces issues including incompatibility with exiting solar cell infrastructure, bulky setup, etc. Yao et al. designed a high efficiency dispersive mirror based on dielectric metasurfaces composed of multiple high-index-contrast gratings (HCG) layers vertically stacked [115]. In their design, each layer has a similar structure with increasing pitch from the top layer to the bottom layer (Fig. 19b). Thus, the operation wavelength increases from top to bottom and this structure can cover the whole sunlight spectrum. Each layer shows a near total reflection in its operation band while longer wavelengths can transmit with negligible loss. Moreover, the chirping periods of each layer are different so the light of each band can be steered into different angles. And it is noteworthy that this structure is polarization independent, which is important in sunlight concentration. This dispersive mirror can replace the secondary mirror of the dome solar concentrator system and combine the function of concentration and spectral splitting. The high efficiency, potentially low cost, and minimal disruption to existing solar energy infrastructure provide this design with opportunities to be accepted by the industry and be widely used.

## Conclusion and outlook

In summary, recent progress in the applications of optical metalenses is reviewed. We introduce some general design principles of metalenses and demonstrate them with examples of experimental work on a wide spectrum. Next, we focus on the correction of chromatic and monochromatic aberrations. A variety of methods have been proposed to deal with the chromatic dispersion or angular dispersion by metalenses. Metalenses that can operate under incident light of multi-wavelengths, with continuous wavelength range and large angles are presented based on these approaches. With all these advances, metasurfaces, with their advantages like flatness, light-weight, CMOS compatible fabrication and small-footprint have exhibited potential to replace (or at least complement) the functionalities of conventional optical diffractive elements. The authors of this paper believe that achromatic, wide FOV and high efficiency metalenses operating in the visible and near infrared will be a game-changer in many applications such as portable and wearable imaging systems, augmented reality and virtual reality glasses, unmanned flying vehicles, automotive, and large-scale solar energy harvesting systems. Nevertheless, many critical problems and challenges are required to be solved for the realization of these possibilities. One of the key topics to be investigated concerns high NA and high efficiency metalenses. To date, few experimental results have been reported to demonstrate a simultaneous realization of high NA and high efficiency though a recent simulation study has been applied to demonstrate a good tradeoff between these two figures (NA = 0.9 with average efficiency ~ 40%) using topology optimization [66]; the results are expected to be verified by experimental works. However, the complexity of fabrication seems to be a barrier to large scale production. Another research subject of current interest relates to tunable metasurfaces, especially the varifocal metalens. Many approaches have been proposed to achieve reconfigurable metalenses that can tune their focal length by using mechanical strain [117, 118], electrical tuning [119, 120], microelectromechanical systems [121], or phase-change materials [122, 123]. However, several key features, such as tuning speed, tuning range and power consumption, for electrically reconfigurable metalenses need to be further improved to compete with current commercial zoom lens cameras.

Metasurfaces lenses are the basis of a research subject that now attracts a large amount of attention and continuous progress will be made in the foreseeable future to tackle the problems mentioned. Emerging methods like inverse design, which enables exploration to the physical limits of nanophotonic devices, will introduce more possibilities and will take this field to the next stage. With all these unparalleled advantages, the authors believe that metalenses possess promising prospects in areas like integrated optics, wearable and portable optical systems where low-cost, miniature footprint and light-weight are highly desirable.
